# Supplementary material for: Substrate promiscuity of key resistance P450s confers clothianidin resistance while increasing chlorfenapyr potency in malaria vectors
Source: Cell Rep. 2024 Jul 31;43(8):114566. doi: 10.1016/j.celrep.2024.114566 (PMC11372441; doi:10.1016/j.celrep.2024.114566)
Supplement: Document S1. Figures S1–S9 and Tables S1–S8 [file mmc1.pdf]

Cell Reports, Volume *43*

Supplemental information

Substrate promiscuity of key resistance P450s confers clothianidin resistance while increasing chlorfenapyr potency in malaria vectors

Magellan Tchouakui, Sulaiman S. Ibrahim, Mersimine K. Mangoua, Riccado F. Thiomela, Tatiane Assatse, Sonia L. Ngongang-Yipmo, Abdullahi Muhammad, Leon J.M. Mugenzi, Benjamin D. Menze, Themba Mzilahowa, and Charles S. Wondji

Supplemental files

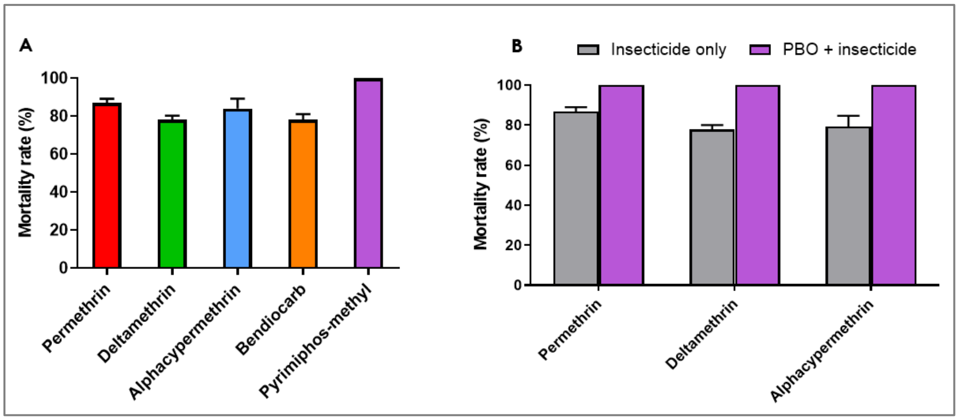

Figure S1: Susceptibility profile of the hybrid strain FG/FZ F_3 to the four main classes of insecticides recommended by WHO: A) Mortality rate after exposure to pyrethroids (permethrin, deltamethrin and alpha-cypermethrin), carbamate (bendiocarb) and organophosphate (pirimiphos-methyl) following 60-min exposure; B) PBO synergist assays with pyrethroids; Data are shown as mean ± SEM.

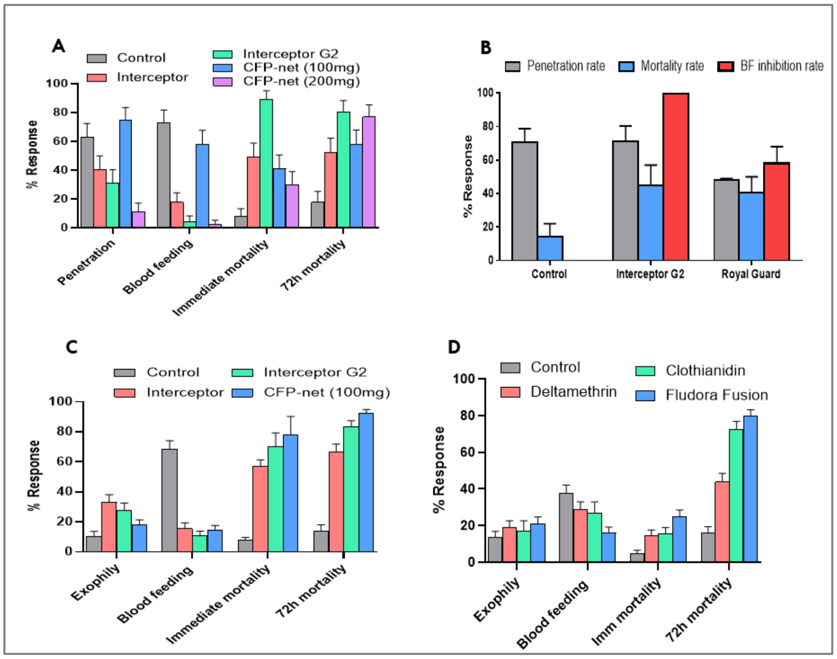

Figure S2: Efficacy of chlorfenapyr and clothianidin-based control tools against *An. funestus* in tunnel assays and experimental hut trials: A) Efficacy of CFP-based nets compared to pyrethroid-only net on the hybrid strain FG/FZ in tunnel tests; B) Efficacy of interceptor G2 and Royal Guard nets on *An. funestus* from Malawi in tunnel tests; C) Efficacy of CFP-based nets compared to pyrethroid-only net on the hybrid strain FG/FZ in EHT; D) Efficacy of the CLTD-based IRS formulations compared to pyrethroid-only IRS product in EHT. Data are shown as mean ± 95% CI.

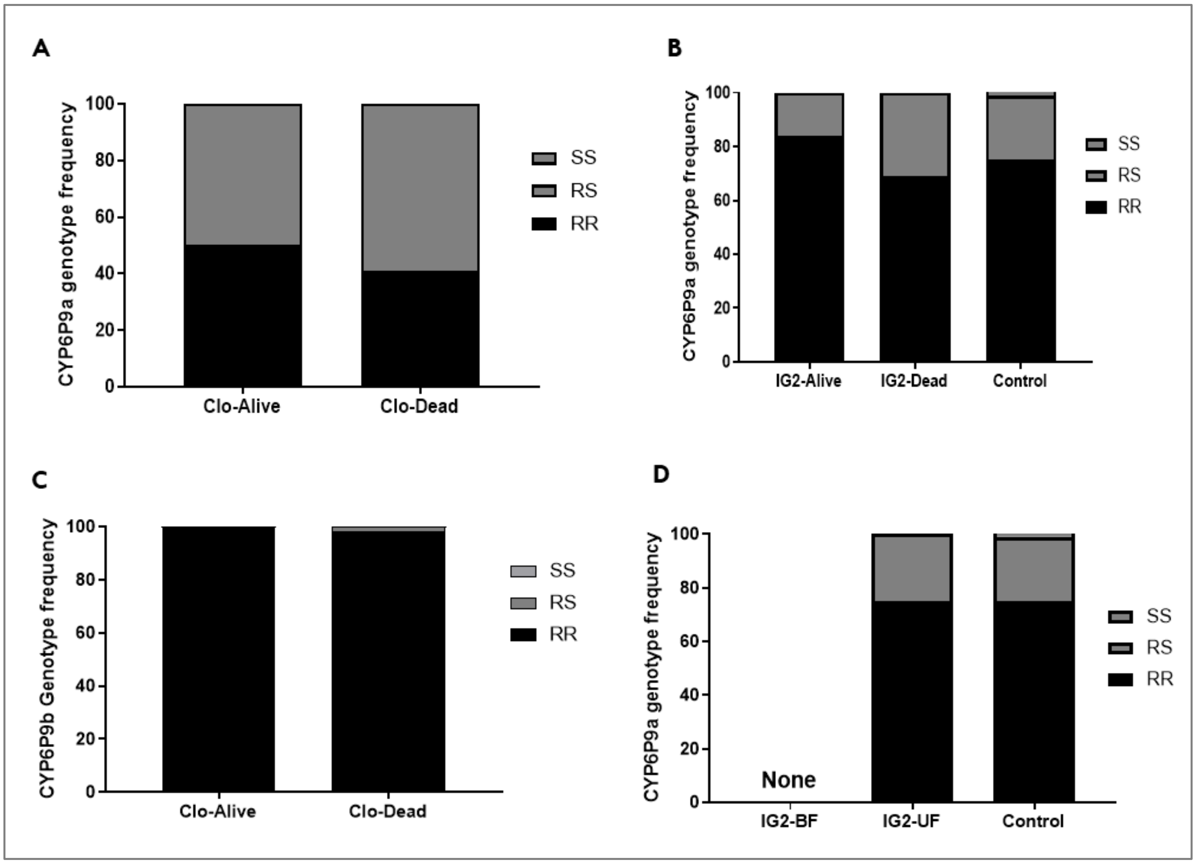

Figure S3: Impact of *CYP6P9a and CYP6P9b* on the ability of field *An. funestus* from Malawi to survive clothianidin and chlorfenapyr exposure. Genotype distribution of CYP6P9a (A) and CYP6P9b (C) between alive and dead after exposure to clothianidin in CDC bottle assays. Genotype distribution of CYP6P9a between dead and alive (B), and blood-fed/unfed (B) after exposure to the CFP-based net IG2 in tunnel test.

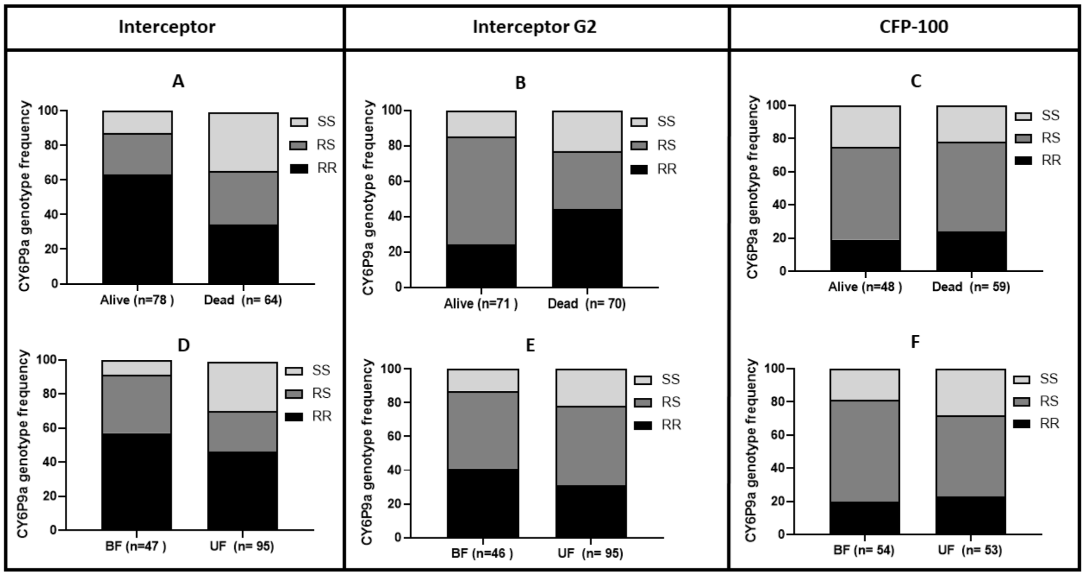

Figure S4: Impact of the *CYP6P9a* on the efficacy of CFP-based nets on the hybrid strain FG/FZ after tunnel tests . Genotype distribution between alive and dead after exposure to Interceptor (A), Interceptor G2 (B) and CFP-100 (C); Genotype distribution between blood fed and unfed after exposure to Interceptor (D), Interceptor G2 (E) and CFP-100 (F);

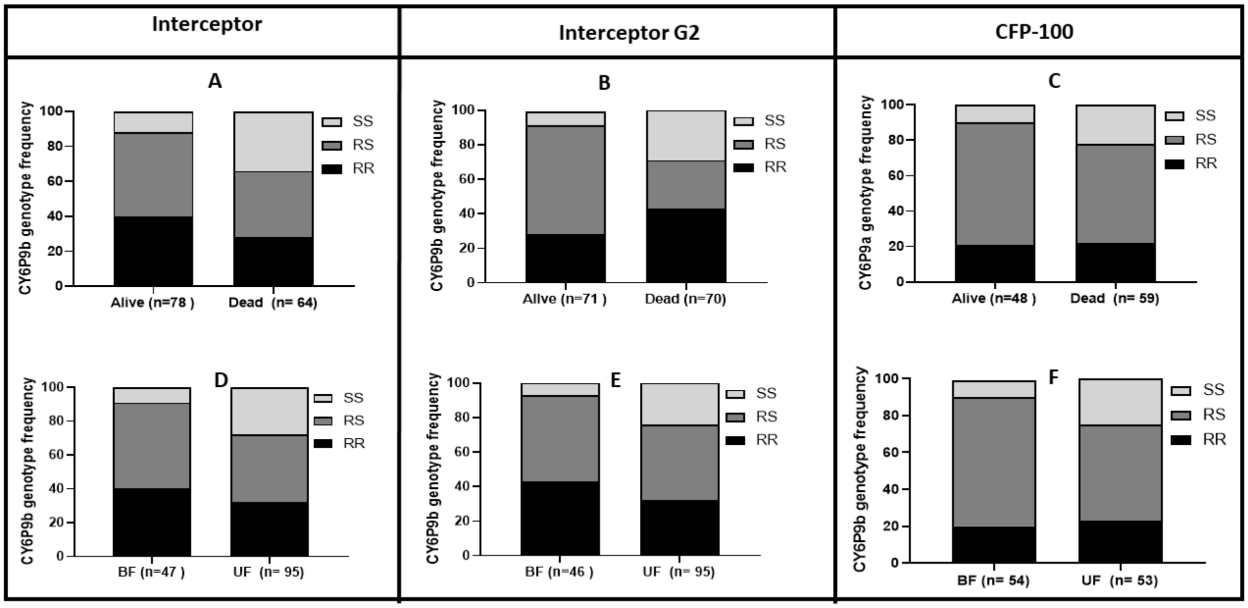

Figure S5: Impact of the *CYP6P9b* on the efficacy of CFP-based nets on the hybrid strain FG/FZ after tunnel tests. Genotype distribution between alive and dead after exposure to Interceptor (A), Interceptor G2 (B) and CFP-100 (C); Genotype distribution between blood fed and unfed after exposure to Interceptor (D), Interceptor G2 (E) and CFP-100 (F);

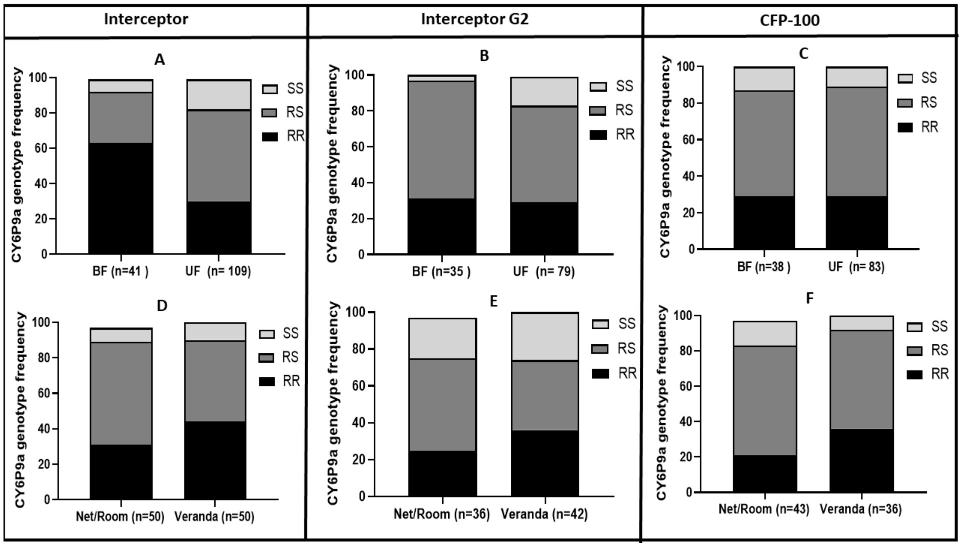

Figure S6: Impact of the C*YP6P9a* on the efficacy of CFP-based nets on the hybrid strain FG/FZ in EHT. Genotype distribution between blood-fed and unfed after exposure to Interceptor (A), Interceptor G2 (B) and CFP-100 (C); Genotype distribution between indoor (Net/Room) and outdoor (veranda) after exposure to Interceptor (D), Interceptor G2 (E) and CFP-100 (F).

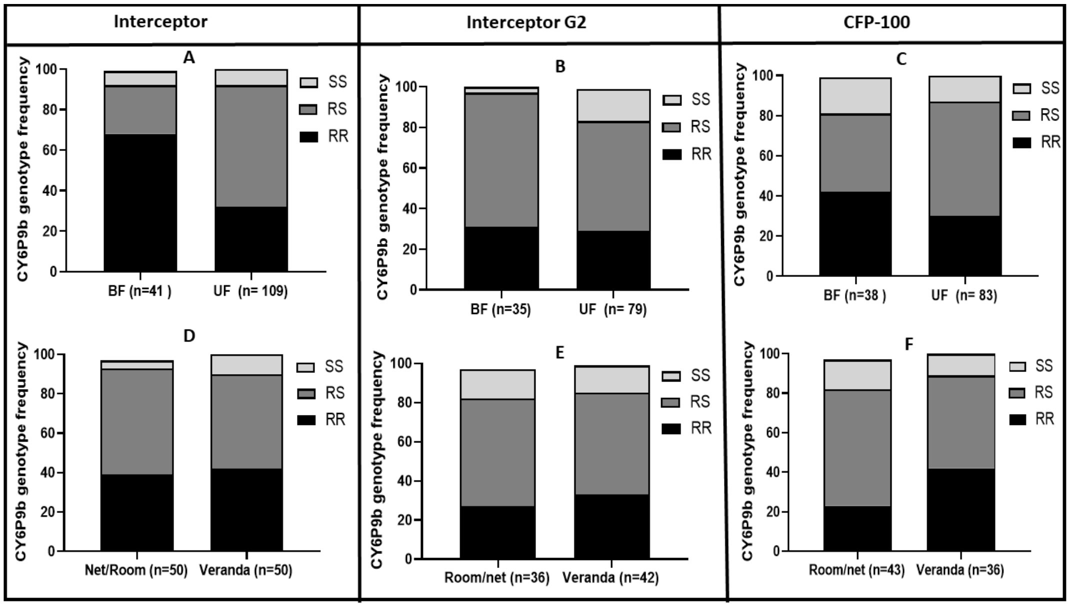

Figure S7: Impact of the *CYP6P9b* on the efficacy of CFP-based nets on the hybrid strain FG/FZ in EHT. Genotype distribution between blood-fed and unfed after exposure to Interceptor (A), Interceptor G2 (B) and CFP-100 (C); Genotype distribution between indoor (Net/Room) and outdoor (veranda) after exposure to Interceptor (D), Interceptor G2 (E) and CFP-100 (F).

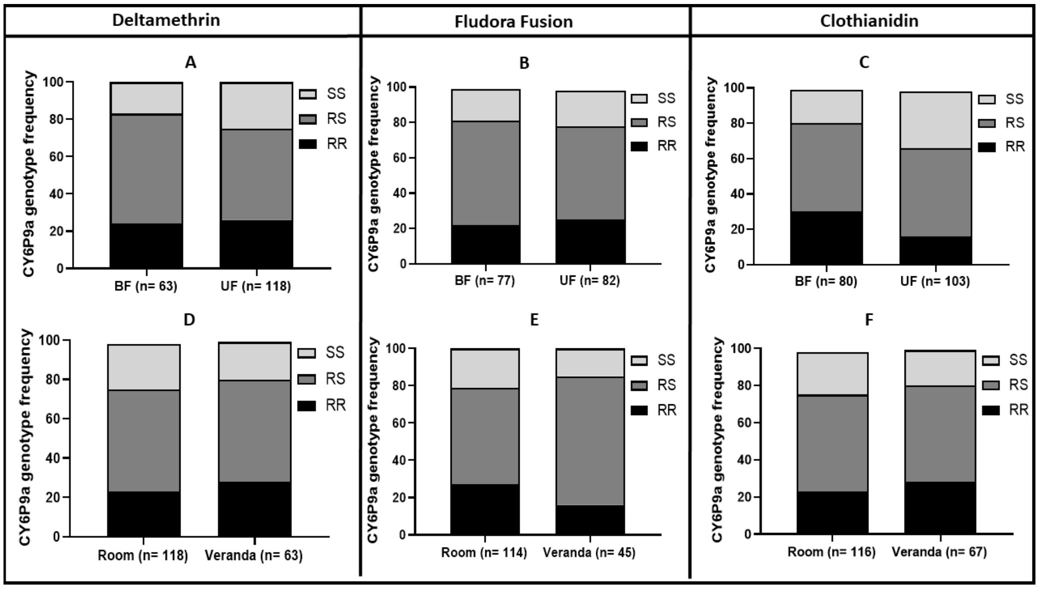

Figure S8: Impact of the *CYP6P9a* on the efficacy of clothianidin-based IRS formulation on the hybrid strain FG/FZ in EHT. Genotype distribution between blood-fed and unfed after exposure to deltamethrin (A), Fludora Fusion (B) and clothianidin (C); Genotype distribution between indoor (Room) and outdoor (veranda) after exposure to deltamethrin (D), Fludora Fusion (E) and clothianidin (F).

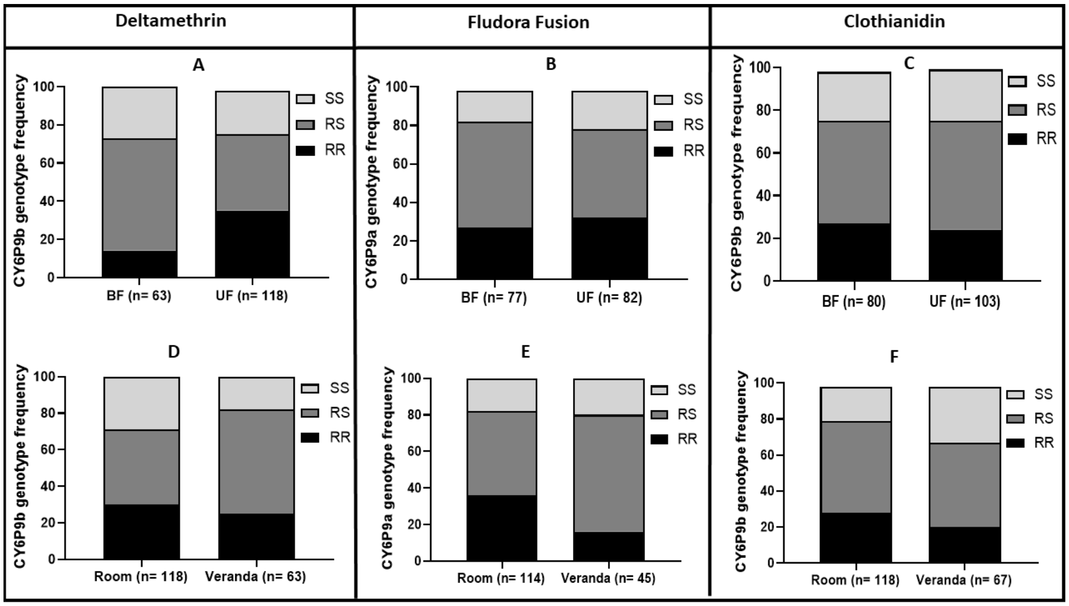

Figure S9: Impact of the *CYP6P9b* on the efficacy of clothianidin-based IRS formulation on the hybrid strain FG/FZ in EHT. Genotype distribution between blood-fed and unfed after exposure to deltamethrin (A), Fludora Fusion (B) and clothianidin (C); Genotype distribution between indoor (Room) and outdoor (veranda) after exposure to deltamethrin (D), Fludora Fusion (E) and clothianidin (F).

Table S1: Correlation between genotypes of the CYP6P9a/b and ability of the hybrid strain FG/FZ to survive CFP exposure in CDC bottle assays

	Odd-Ratio	Confidence interval	*P*-value
**Genotypes at *CYP6P9a* locus**			
**RR vs SS**	0.1	0.06 - 0.3	<0.0001
**RS vs SS**	0.7	0.3 - 1.7	0.7
**RR vs RS**	0.1	0.09 - 0.3	<0.0001
**R vs S**	0.7	0.4 - 1.3	0.4
**Genotypes at *CYP6P9b* locus**			
**RR vs SS**	0.2	0.1 - 0.5	0.0003
**RS vs SS**	0.7	0.3 -1.5	0.09
**RR vs RS**	0.3	0.2 - 0.7	0.002
**R vs S**	0.6	0.9 - 2.7	0.1

OR: odd-ratio; CI: confidence interval; RR: homozygote resistant; RS: heterozygote; SS: homozygote susceptible.

Table S2: Impact of CYP6P9a and b on the ability of the hybrid strain FG/FZ to survive clothianidin exposure in CDC bottle assay

	Odd-Ratio	Confidence interval	*P*-value
**Genotypes at *CYP6P9a* locus**			
**RR vs SS**	7.5	2.1-27.5	0.001
**RS vs SS**	3.5	1.1-11.5	0.02
**RR vs RS**	2.1	0.9-5.1	0.06
**R vs S**	2.2	1.2-3.8	0.005
**Genotypes at *CYP6P9b* locus**			
**RR vs SS**	7.1	1.9-26.1	0.002
**RS vs SS**	3	0.9-9.9	0.05
**RR vs RS**	2.4	0.9-5.6	0.04
**R vs S**	2.2	1.2-3.8	0.005

OR: odd-ratio; CI: confidence interval; RR: homozygote resistant; RS: heterozygote; SS: homozygote susceptible.

Table S3: Performance of clothianidin-based IRS against F_3 hybrid FG/FZ *An. funestus* mosquitoes

**Outcomes**	**Control**	**Deltamethrin (25 mg/m2)**	**Clothianidin (200 mg/m2)**	**Fludora® Fusion (225 mg/m2)**
**females caught**	339	447	408	471
**%Exophily (95% CI)**	13.67 (10.45-16.88) [a]	19.02 (15.38-22.65) [b]	17.16 (13.50-20.82) [b]	21.02 (17.34-24.70) [b]
**%Blood fed (95% CI)**	37.59 (33.05-42.12) [c]	28.64 (24.44-32.83) [b]	26.72 (22.42-31.01) [b]	15.92 (12.62-19.23) [a]
**% Immediate mortality (95% CI)**	4.56 (2.61-6.51) [a]	14.32 (11.07-17.56) [b]	15.44 (11.93-18.95) [b]	24.63 (20.74-28.52) [c]
**%Mortality (72 h) (95% CI)**	15.95 (12.52-19.37) a	43.85 (39.25-48.45) [b]	72.55 (68.22-76.88) [c]	79.62 (75.98-83.26) [c]

Table S4: Correlation between genotypes at the *CYP6P9a/b* locus and the ability to survive/blood feed in the presence of various nets in tunnel tests

**Genotype combination**	**Interceptor**			**Interceptor G2**			**CFP-100**		
	**OR**	**CI**	***P*-value**	**OR**	**CI**	***P*-value**	**OR**	**CI**	***P*-value**
	**Impact of *CYP6P9a* mortality**								
**RR vs SS**	123	18-1273	<0.0001	1.7	0.7-4.6	0.3	0.8	0.3-1.9	0.8
**RS vs SS**	66	10-684	<0.0001	1.1	0.5-2.3	0.8	2.2	1.2-4.5	0.01
**RR vs RS**	1.8	0.9-3.8	0.1	1.5	0.7-3.3	0.2	0.3	0.1-0.8	0.02
**R vs S**	4.3	2.3-7.6	<0.0001	1.2	0.7-2.2	0.4	1.1	0.6-1.8	0.8
	**Impact of *CYP6P9b* mortality**								
**RR vs SS**	4.04	1.8-8.7	0.0006	2.3	0.9-5.7	0.08	2.1	0.8-5.1	0.1
**RR vs RS**	1.1	0.5-2.1	0.7	0.2	0.1-0.5	0.0002	0.7	0.3-1.5	0.4
**RS vs SS**	3.5	1.5-8.04	0.001	8.1	3.2-19.4	<0.0001	2.7	1.1-6.1	0.01
**R vs S**	2.0	1.1-3.4	0.02	1.1	0.6-1.9	0.7	1.2	0.6-2.1	0.5
	**Impact of *CYP6P9a* on blood feeding success**								
**RR vs SS**	0.8	0.4-1.6	0.7	1.3	0.7- 2.4	0.3	0.6	0.3-1.4	0.3
**RS vs SS**	3.9	1.7-9.7	0.001	2.2	0.9-5.3	0.06	1.2	0.5- 2.9	0.6
**RR vs RS**	4.5	1.8-10.6	0.0008	1.6	0.7-3.7	0.2	1.8	0.9-3.6	0.1
**R vs S**	2.06	1.1-3.6	0.02	1.5	0.8-2.6	0.1	1.1	0.6-2.06	0.6
	**Impact of *CYP6P9b* on blood feeding success**								
**RR vs SS**	3.8	1.5-8.9	0.002	4.6	1.7-11.4	0.0013	2.4	0.9-6.1	0.09
**RR vs RS**	0.9	0.5-1.8	>0.9	1.1	0.6- 2.1	0.6	0.6	0.3-1.2	0.2
**RS vs SS**	3.9	1.6-9.2	0.001	3.8	1.5-9.7	0.0035	3.7	1.6-9.1	0.002
**R vs S**	1.7	1-3.1	0.06	1.8	1-3.2	0.05	1.3	0.7-2.3	0.3

OR: odd-ratio; CI: confidence interval; CFP: chlorfenapyr; RR: homozygote resistant; RS: heterozygote; SS: homozygote susceptible.

Table S5: Correlation between genotypes at the CYP6P9a/b locus and the ability to survive exposure/blood feed in the presence of various nets in experimental huts

Genotype combination	Interceptor			Interceptor G2			CFP-100		
	OR	CI	P-value	OR	CI	P-value	OR	CI	P-value
	**Impact of *CYP6P9a* mortality**								
**RR vs SS**	47,5	7,6-497,8	<0,0001	13,2	2,2-44,3	0,003	0,3	0,1-1,3	0,1
**RS vs SS**	27,1	4,5-285,4	<0,0001	25,00	3,9-265,6	<0,0001	3,5	1,3-9,7	0,01
**RR vs RS**	1,7	0,9-3,1	0,09	0,5	0,2-0,9	0,05	0,1	0,04-0,2	<0,0001
**R vs S**	3,0	1,6-5,7	0,0004	1,7	1,-3,06	0,06	0,6	0,3-1,1	0,2
	**Impact of *CYP6P9b* mortality**								
**RR vs SS**	25,2	4,2-272,4	<0,0001	13,2	2,2-144,3	0,003	2,2	0,7-6,4	0,2
**RR vs RS**	2,2	1,2-4,04	0,008	0,5	0,2-0,9	0,05	0,6	0,3-1,1	0,1
**RS vs SS**	11,2	1,6-121,3	0,004	25	3,9-265,6	<0,0001	3,5	1,3-9,5	0,01
**R vs S**	2,4	1,3-4,6	0,005	1,2	0,7-2,2	0,4	1,1	0,6-1,9	0,7
	**Impact of *CYP6P9a* on blood feeding success**								
**RR vs SS**	5,1	1,8-12,7	0,0009	5,7	1,6-19,6	0,007	0,8	0,3-2,1	0,8
**RS vs SS**	1,3	0,5-3,4	0,6	6,5	1,8- 21,7	0,002	0,8	0,3-1,9	0,8
**RR vs RS**	3,7	1,9-7,1	<0,0001	0,8	0,4-1,6	0,7	1,03	0,5-1,9	>0,9
**R vs S**	2,7	1,5-5,2	0,001	1,3	0,7-2,4	0,3	0,9	0,5-1,712	>0,9
	**Impact of *CYP6P9b* on blood feeding success**								
**RR vs SS**	2,4	0,76,6	0,1	5,7	1,6-19,60	0,007	1,01	0,4-2,3	>0,9
**RR vs RS**	5,3	2,7-9,7	<0,0001	0,8	0,4-1,6	0,7	2,04	1,07-3,7	0,02
**RS vs SS**	0,4	0,1-1,3	0,2	6,5	1,8-21,7	0,002	0,4	0,2-1,09	0,1
**R vs S**	2,4	1,2-4,5	0,007	1,3	0,7-2,4	0,3	1,1	0,6-2,04	0,6

OR: odd-ratio; CI: confidence interval; CFP: chlorfenapyr; RR: homozygote resistant; RS: heterozygote; SS: homozygote susceptible.

Table S6: Correlation between genotypes at the CYP6P9a/b locus and the ability to survive exposure/blood feed in the presence of various IRS treatments in experimental huts

Genotype combination	Deltamethrin			Clothianidin			Fludora		
	OR	CI	*P*-value	OR	CI	P-value	OR	CI	*P*-value
	**Impact of *CYP6P9a* mortality**								
**RR vs SS**	2.5	1.3-4.7	0.001	0.7	0.4-1.5	0.2	1.2	0.6-2.3	0.3
**RS vs SS**	11.4	3.2-41.3	<.0001	1.1	0.5-2.4	0.5	2	0.8-5.2	0.1
**RR vs RS**	4.4	1.2-16.4	0.01	1.4	0.7-3.0	0.1	1.6	0.7-3.9	0.2
**R vs S**	3.4	1.7-6.5	0.0001	1	0.6-1.8	0.5	1.3	0.7-2.3	0.2
	**Impact of *CYP6P9b* on mortality**								
**RR vs SS**	11.4	3.2-41.3	<.0001	1.5	0.5-2.4	0.3	1.1	0.8-5.2	0.1
**RR vs RS**	2.5	1.3-4.7	0.001	0.7	0.4-1.5	0.6	1.2	0.6-2.3	0.3
**RS vs SS**	4.4	1.2-16.4	0.01	2.05	0.7-3.0	0.05	1.6	0.7-3.9	0.2
**R vs S**	3.4	1.7-6.5	0.0001	1	0.6-1.8	0.5	1.3	0.7-2.3	0.2
	**Impact of *CYP6P9a* on blood-feeding success**								
**RR vs SS**	0.2	0.1-0.4	<.0001	1.2	0.6-2.3	0.4	0.4	0.2-0.7	**0.004**
**RS vs SS**	0.3	0.1-0.8	0.02	1.6	0.7-3.6	0.1	0.7	0.3-1.8	0.3
**RR vs RS**	1.5	0.5-4.15	0.6	1.4	0.7-2.8	0.7	2	0.8-4.6	0.7
**R vs S**	0.3	0.2-0.6	0.0007	0.7	0.4-1.4	0.2	0.7	0.4-1.4	0.2
	**Impact of *CYP6P9b* on blood-feeding success**								
**RR vs SS**	0.3	0.1-0.8	0.01	1.6	0.7-3.6	0.1	1.4	0.3-1.8	0.3
**RR vs RS**	0.27	0.1-0.6	<.0001	1.2	0.6-2.3	0.4	0.7	0.2-0.7	**0.004**
**RS vs SS**	1.2	0.6-2.4	0.6	0.98	0.7-2.8	0.9	2	0.8-4.6	0.7
**R vs S**	0.3	0.2-0.6	0.0007	0.7	0.4-1.4	0.2	0.7	0.4-1.4	0.2

OR: odds ratio; CI: confidence interval; RR: homozygote resistant; RS: heterozygote; SS: homozygote susceptible.

Table S7: *Combined impact of CYP6P9a and CYP6P9b on the mortality and blood feeding of* the hybrid strain FG/FZ *after exposure to various nets in tunnel assays*

**Interceptor**						
	**Mortality**			**Blood feeding**		
	**OR**	**CI**	**P-value**	**OR**	**CI**	**P-value**
**RR/RR vs RS/RS**	1.7	0.8-3.7	0.1	0.9	0.4-2	>0.9
**RR/RR vs SS/SS**	8.06	2.7-20.7	<0.0001	3.2	1.2-7.6	0.01
**RR/RR vs SS/RR**	0.6	0.1-2.8	0.6	5.3	0.7-66.7	0.1
**RR/RR vs SS/RS**	0.3	0.02-2.08	0.3	4	0.5-53.1	0.3
**RS/RS vs SS/SS**	4.6	1.6-12.1	0.004	3.3	1.2-8.2	0.01
**RS/RS vs RS/SS**	1.4	0.15-21.1	>0.9	2.7	0.29-40.6	0.5
**RS/RS vs RR/SS**	7.1	0.8-86.4	0.08	0.1	0.01-1.2	0.1
**Interceptor G2**						
	**OR**	**CI**	**P-value**	**OR**	**CI**	**P-value**
**RR/RR vs RS/RS**	0.1	0.06-0.3	<0.0001	1.6	0.7-3.3	0.2
**RR/RR vs SS/SS**	0.8	0.32.4	>0.9	5.9	1.6-17.4	0.002
**RR/RR vs SS/RR**	0.2	0.01-5.6	0.4	1.1	0.05-23.2	>0.9
**RR/RR vs SS/RS**	0.03	0.003-0.2	0.0006	0.1	0.01-1.002	0.07
**RS/RS vs SS/SS**	5.5	2.07-13.7	0.0004	3.6	1.1-10.7	0.02
**RS/RS vs RS/SS**	1.6	0.08-32.7	>0.9	0.7	0.03-14.4	>0.9
**RS/RS vs RR/SS**	0.1	0.01-1	0.1	0.9	0.1-4.6	>0.9
**CFP-100**						
	**OR**	**CI**	**P-value**	**OR**	**CI**	**P-value**
**RR/RR vs RS/RS**	0.9	0.4-1.9	0.8	0.6	0.3-1.3	0.3
**RR/RR vs SS/SS**	1.7	0.6-4.7	0.3	2.5	0.8-7.01	0.1
**RR/RR vs SS/RR**	1.7	0.1-27.2	>0.9	1.5	0.16-23.03	>0.9
**RR/RR vs SS/RS**	0.05	0.005-0.4	0.001	0.1	0.02-0.6	0.02
**RS/RS vs SS/SS**	1.9	0.8-4.5	0.1	4	1.6-10.02	0.002
**RS/RS vs RR/SS**	/	/	/	1.7	0.19-25.3	>0.9

Table S8: *Combined impact of CYP6P9a and CYP6P9b on the mortality and blood feeding of* the hybrid strain FG/FZ *after exposure to various nets in EHT*

**Interceptor**						
	**Mortality**			**Blood feeding**		
	**OR**	**CI**	**P-value**	**OR**	**CI**	**P-value**
**RR/RR vs RS/RS**	1.8	1.01-3.5	0.05	4.8	2.4-9.6	<0.0001
**RR/RR vs SS/SS**	25.04	4.06-271.2	<0.0001	2.1	0.7-6.3	0.2
**RR/RR vs SS/RS**	25.04	4-271.2	<0.0001	21.7	3.2-240	0.0002
**RS/RS vs SS/SS**	13.3	2.2-145	0.002	0.4	0.1-1.3	0.2
**RS/RS vs RS/SS**	0.9	0.05-18.9	>0.9	/	/	/
**RS/RS vs RR/SS**	/	/	/	2.2	0.1-43.05	0.5
**Interceptor G2**						
	**OR**	**CI**	**P-value**	**OR**	**CI**	**P-value**
**RR/RR vs RS/RS**	0.50	0.2-0.97	0.05	2.5	1.2-5.03	0.01
**RR/RR vs SS/SS**	13.8	2.2-151.2	0.003	1.1	0.4-3.2	0.8
**RR/RR vs SS/RR**	1.6	0.1-24.5	>0.9	4.9	0.6-65.8	0.2
**RR/RR vs SS/RS**	2.4	0.6-9.07	0.3	0.5	0.1-1.9	0.3
**RS/RS vs SS/SS**	27.2	4.2-290	<0.0001	0.4	0.1-1.1	0.1
**CFP-100**						
	**OR**	**CI**	**P-value**	**OR**	**CI**	**P-value**
**RR/RR vs RS/RS**	0.1	0.05-0.3	<0.0001	1.3	0.6-2.7	0.4
**RR/RR vs SS/SS**	0.4	0.1-1.5	0.3	0.8	0.3-2.1	0.7
**RR/RR vs SS/RS**	0.2	0.01-4.8	0.3	0.2	0.02-2.04	0.3
**RS/RS vs SS/SS**	3.02	1.03-8.7	0.04	0.6	0.2-1.5	0.3
**RS/RS vs RS/SS**	6.04	0.9-74.9	0.1	0.2	0.04-1.2	0.1
**RS/RS vs RR/SS**	0.6	0.03-12.8	>0.9	4.9	0.7-65.5	0.2
