# Supplementary material for: Substrate promiscuity of key resistance P450s confers clothianidin resistance while increasing chlorfenapyr potency in malaria vectors
Source: Cell Rep. 2024 Jul 31;43(8):114566. doi: 10.1016/j.celrep.2024.114566 (PMC11372441; doi:10.1016/j.celrep.2024.114566)
Supplement: Document S2. Article plus supplemental information [file mmc2.pdf]

Article

Cell Reports

Substrate promiscuity of key resistance P450s confers clothianidin resistance while increasing chlorfenapyr potency in malaria vectors

Graphical abstract

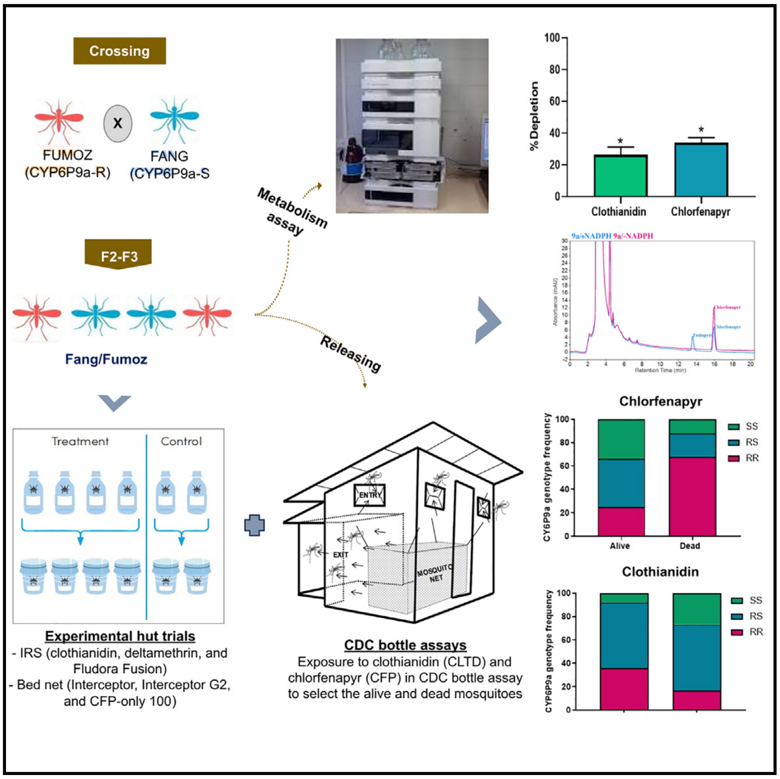

Highlights

- Pyrethroid resistance CYP6P9a_R/-b_R markers drive clothianidin resistance in *An. funestus*
- Double homozygote genotypes (RR) exhibit significantly higher clothianidin resistance
- There is increased chlorfenapyr susceptibility in individuals with the above markers
- The above observations are confirmed with *in vivo* and *in vitro* functional tools

Authors

Magellan Tchouakui, Sulaiman S. Ibrahim, Mersimine K. Mangoua, ..., Benjamin D. Menze, Themba Mzilahowa, Charles S. Wondji

Correspondence

magellan.tchouakui@crid-cam.net (M.T.), charles.wondji@lstmed.ac.uk (C.S.W.)

In brief

Tchouakui et al. use P450 DNA-based markers of pyrethroid resistance in *Anopheles funestus*, coupled with extensive *in vivo* and *in vitro* functional validation, to demonstrate that the proficient pyrethroids metabolizing P450s, CYP6P9a/-b, are reducing neonicotinoid efficacy in malaria vectors while exacerbating the potency of chlorfenapyr. This will facilitate evidence-based control and resistance management.

Tchouakui et al., 2024, Cell Reports *43*, 114566
August 27, 2024 © 2024 The Authors. Published by Elsevier Inc.
https://doi.org/10.1016/j.celrep.2024.114566

Cell Reports

CellPress
OPEN ACCESS

Article

Substrate promiscuity of key resistance P450s confers clothianidin resistance while increasing chlorfenapyr potency in malaria vectors

Magellan Tchouakui,[1,8,*] Sulaiman S. Ibrahim,[1,2,3] Mersimine K. Mangoua,[1] Riccado F. Thiomela,[1,4] Tatiane Assatse,[1,4] Sonia L. Ngongang-Yipmo,[1,4] Abdullahi Muhammad,[3,5] Leon J.M. Mugenzi,[1] Benjamin D. Menze,[1] Themba Mzilahowa,[6] and Charles S. Wondji[1,3,7,*]

[1]Centre for Research in Infectious Diseases (CRID), Medical Entomology Department, P.O. Box 13501, Yaoundé, Cameroon
[2]Department of Biochemistry, Bayero University, PMB 3011, Kano, Nigeria
[3]Department of Vector Biology, Liverpool School of Tropical Medicine, Pembroke Place, Liverpool L35QA, UK
[4]Parasitology and Ecology Laboratory, Department of Animal Biology and Physiology, Faculty of Science, University of Yaoundé 1, P.O. Box 812, Yaoundé, Cameroon
[5]Centre for Biotechnology Research, Bayero University, PMB 3011, Kano, Nigeria
[6]Malaria Alert Centre (MAC), Kamuzu University of Health Sciences (KUHeS), Entomology Department, P.O. Box 265, Blantyre, Malawi
[7]International Institute of Tropical Agriculture (IITA), P.O. Box 2008, Yaoundé, Cameroon
[8]Lead contact
*Correspondence: magellan.tchouakui@crid-cam.net (M.T.), charles.wondji@lstmed.ac.uk (C.S.W.)
https://doi.org/10.1016/j.celrep.2024.114566

SUMMARY

Novel insecticides were recently introduced to counter pyrethroid resistance threats in African malaria vectors. To prolong their effectiveness, potential cross-resistance from promiscuous pyrethroid metabolic resistance mechanisms must be elucidated. Here, we demonstrate that the duplicated P450s *CYP6P9a/-b*, proficient pyrethroid metabolizers, reduce neonicotinoid efficacy in *Anopheles funestus* while enhancing the potency of chlorfenapyr. Transgenic expression of *CYP6P9a/-b* in *Drosophila* confirmed that flies expressing both genes were significantly more resistant to neonicotinoids than controls, whereas the contrasting pattern was observed for chlorfenapyr. This result was also confirmed by RNAi knockdown experiments. *In vitro* expression of recombinant *CYP6P9a* and metabolism assays established that it significantly depletes both clothianidin and chlorfenapyr, with metabolism of chlorfenapyr producing the insecticidally active intermediate metabolite tralopyril. This study highlights the risk of cross-resistance between pyrethroid and neonicotinoid and reveals that chlorfenapyr-based control interventions such as Interceptor G2 could remain efficient against some P450-based resistant mosquitoes.

INTRODUCTION

Reduction in malaria transmission in Africa before 2015 was predominantly due to increased use of pyrethroid-impregnated bed nets.[1] Out of the 663 million malaria cases averted in sub-Saharan Africa between 2001 and 2015, it was estimated that nearly 80 percent were due to the use of insecticide-treated nets (ITNs) and indoor residual spraying (IRS).[1] Despite the gains achieved by cost-effective vector control interventions, multiple factors threaten future progress among which insecticide resistance, residual transmission, and invasive vector species[2] take the front seat. Among all these factors, the continuous spreading/escalation of resistance to pyrethroid insecticides in major malaria vectors[3–5] is a big obstacle to vector control.[6] Since 2010, resistance to at least one class of insecticide has been reported in 61 countries arising mainly through increased expression of detoxification genes (metabolic resistance), including cytochrome P450s, glutathione S-transferases, and carboxylesterases.[7,8] This has been a major contributor to vector control failure leading to malaria resurgence in recent years.[6] Insecticide resistance can also arise through insecticide target site modifications, which reduce insecticide binding, either in the voltage-gated sodium channels, e.g., the knockdown resistance (*kdr*) mutations,[9] or in the acetylcholine esterase receptor (Ace-1) gene[10]; behavioral avoidance, which reduces contact with the insecticides[11]; and reduced insecticide penetration through increased production of cuticular hydrocarbons.[12]

Consequently, it is imperative to design novel molecules with completely different modes of action to mitigate the growing challenge of pyrethroid resistance. To delay or reverse the spread of resistance, novel insecticides are gradually being introduced by manufacturers and recommended by WHO for vector control, to be used either in rotation or in mixture with existing insecticides.[13] Among these novel insecticides, chlorfenapyr (CFP), a pyrrole insecticide that works by disrupting respiratory pathways and proton gradients in mitochondria,[14,15] and the neonicotinoids

This is an open access article under the CC BY license (http://creativecommons.org/licenses/by/4.0/).

targeting nicotinic acetylcholine receptors (nAChRs)[16] are new recommended insecticides by WHO for vector control. These insecticide chemistries have been presented as good alternatives as they have different mechanisms of action and unique targets.[17,18] Interceptor G2 (IG2) net treated with alpha-cypermethrin and CFP is one example of a long-lasting dual insecticide mixture net that has shown great promise at controlling pyrethroid-resistant malaria vectors in randomized cluster control trials (RCTs) conducted in Benin and Tanzania[19–21] and several experimental hut trials (EHTs) across the continent.[21–24] The new insecticide formulation combining the neonicotinoid clothianidin (CLTD) and the pyrethroid deltamethrin (8:1 w/w) under the brand name Fludora Fusion developed by Bayer (Bayer CropScience, Monheim, Germany) for IRS as a tool for insecticide resistance management has also demonstrated its high efficacy against various malaria vectors, including pyrethroid-resistant populations.[17,18] As these new products are introduced, it is vital to evaluate their efficacy against pyrethroid-resistant mosquitoes, notably populations in which P450-based resistance mechanism is predominant, and to investigate the potential positive/negative impact of P450-resistance markers on their performance.

Ample evidence, using population genetics/genomics, and functional genomics have established that allelic variants of *CYP6P9a* and *CYP6P9b*[25,26] (hereby after *CYP6P9a*/-*b*) are major drivers of pyrethroid resistance in *An. funestus* in a large swarth of Africa. Significant progress has been made in recent years with the detection of the first P450-based molecular markers for *An. funestus* CYP6P9a[27] and CYP6P9b[28] and for the 6.5-kb structural variant insertion acting as an enhancer for both *CYP6P9a* and *CYP6P9b* expression.[29] Despite this major achievement, the direct phenotypic impact of major pyrethroid resistance P450 genes on the efficacy of novel insecticides such as CFP and clothianidin remains relatively unknown in malaria vectors, although some studies have explored this question using heterologous expression assays.[30] In this study, we used recently detected DNA-based markers of P450-linked pyrethroid resistance in *An. funestus*, coupled with extensive *in vivo* and *in vitro* function validation to directly establish the impact of the major pyrethroid resistance P450s on novel insecticides with the goal of informing control programs of the risk of cross-resistance or to highlight the potential antagonistic effect that could boost the efficacy of new-generation insecticides. We observed that the duplicated CYP6P9a/b pyrethroid-resistance genes are driving cross-resistance to clothianidin but, in contrast, significantly boosting the insecticidal efficacy of CFP.

RESULTS

Susceptibility profile of *An. funestus* to chlorfenapyr and neonicotinoids

The Malawi field *An. funestus* was fully susceptible to CFP (mortality = 100%), regardless of the solvent used (Figure 1A). This population was also fully susceptible to the neonicotinoids clothianidin and imidacloprid when using acetone + vegetable oil ester (MERO) as a solvent, with a mortality rate of 100% after a holding period of 24 h (Figure 1B). However, when dissolved in acetone alone, a mortality rate of 58.4% ± 8.5% was observed for clothianidin and 34.8% ± 2.0% for imidacloprid (Figure 1B), thus exhibiting a reduced susceptibility. In this population, we noticed that marked aggravation of pyrethroid resistance between 2014 and 2021 was partly linked with increased expression of CYP6P9a/b-P450 alleles.[31]

Susceptibility profiling of the hybrid strain FANGG/FUMOZ to chlorfenapyr, neonicotinoids, and other insecticide classes

The hybrid strain FANG/FUMOZ was fully susceptible to the diagnostic dose (DD) of CFP (100 μg/ml), and dose-response tests revealed that the LC_{50} is obtained with 20 μg/ml of CFP and the LT_{50} obtained after 15-min exposures to the DD (Figures 1C and 1D). Partial recovery of susceptibility was observed when mosquitoes were pre-exposed to PBO and later to a sub-lethal dose of CFP (mortality = 58.2% for 20 μg/ml CFP only vs. 70.7% for pyperonyl butoxide (PBO) + 20 μg/ml CFP; p = 0.06) (Figure 1E). Resistance to neonicotinoids was observed in this strain when the insecticides were diluted in acetone only with mortality rates of 40.3% ± 13.2% for clothianidin, 43.3% ± 11.4% for imidacloprid, and 41.4% ± 8.5% for acetamiprid. Interestingly, partial recovery of susceptibility was obtained with PBO pre-exposure for all three neonicotinoid insecticides (55.3%–64.4% mortality, $p > 0.05$) indicating the contribution of P450s to neonicotinoid resistance (Figure 1F). However, full susceptibility was observed when MERO was added to the solvent with 100% mortality recorded 24 h post exposure (Figure 1F). A moderate pyrethroid resistance was observed with mortality rates of 86.9% ± 2.2%, 77.9% ± 2.2%, and 83.9% ± 5.3% 24 h after exposure to 1× permethrin (0.75%), 1× deltamethrin (0.05%) and 1× alpha-cypermethrin (0.05%), respectively (Figure S1). This hybrid strain was also resistant to the carbamate, bendiocarb (1×, 0.1%), with a mortality rate of 78.1% ± 2.9%, but fully susceptible to the organophosphate pyrimiphos-methyl 1× (mortality = 100%) (Figure S1). PBO pre-exposure induced full susceptibility to all the pyrethroids tested (Figure S1), confirming the role of CYP450s as the major contributors to pyrethroid resistance in this strain.

Impact of CYP6P9a_R/-b_R markers on the ability to survive chlorfenapyr exposure in CDC bottle assays

Mosquitoes with contrasting phenotypes (alive and dead) after 20-min exposure to 100 μg/mL CFP were used to establish the impact of CYP6P9a_R/-b_R markers on CFP susceptibility. Genotyping of 32 alive and 40 dead females after exposure to 20 μg/ml CFP revealed that homozygote-resistant individuals for *CYP6P9a* marker (CYP6P9a_RR) had a significantly higher mortalities upon CFP exposure, compared to the individuals carrying the homozygote susceptible allele (odds ratio [OR] = 0.1; $p < 0.0001$) (Figure 2A; Table S1). An additive effect of this disadvantageous property of *CYP6P9a* was seen in the homozygote pyrethroid-resistant mosquitoes, which died significantly more than heterozygotes (OR = 0.1; $p < 0.0001$) (Table S1), with the frequency of the dead mosquitoes correlating with possessing the CYP6P9a_R allele, and alive mosquitoes being predominantly CYP6P9a_S allele carriers (Figure 2B; Table S1). For *CYP6P9b*, higher mortality was also obtained in the pyrethroid-resistant homozygote RR mosquitoes upon CFP exposure compared to the homozygote susceptible mosquitoes (OR = 0.2; p = 0.0003) and

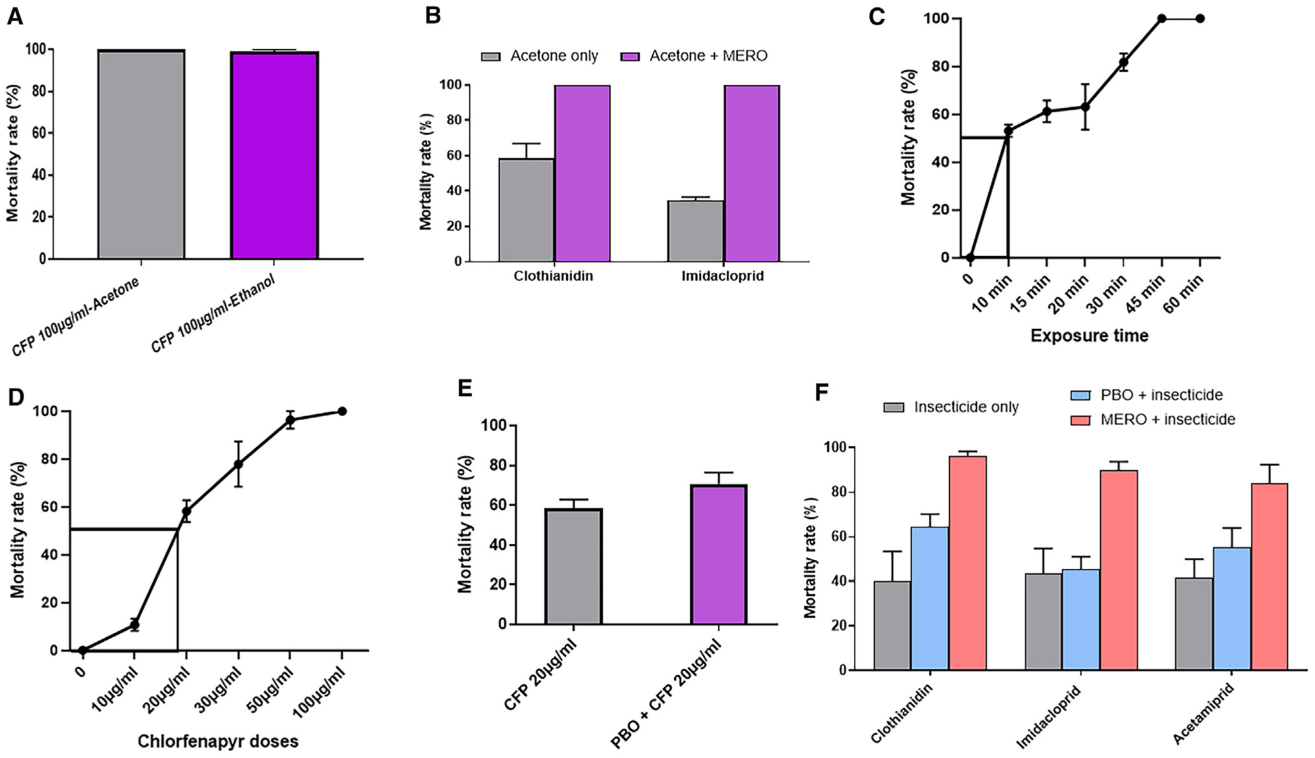

Figure 1. Susceptibility profile of the *An. funestus* from Malawi and the hybrid strain FG/FZ (F_3) to neonicotinoid insecticides and chlorfenapyr

(A–F) Mortality rate of F_1 progeny from field-collected *An. funestus* in Malawi after exposure to chlorfenapyr (A); clothianidin and imidacloprid diluted in acetone only and acetone + MERO (B); time-response mortality after exposure of FG/FZ (F_3) to 100 µg/mL CFP (C); dose-response results of FG/FZ (F_3) after exposure to CFP (D); pyperonyl butoxide (PBO) synergist assay with 20 µg/mL CFP (E); and mortality rate after exposure to PBO/MERO + neonicotinoids (F). In this figure, values represent the mean mortality of 4–5 biological replicates, and error bars represent ±SE of mean. χ^2 test was used to discern significant differences.

heterozygotes (OR = 0.3; p = 0.002) (Figures 2C and 2D; Table S1). Moreover, this negative impact of these two P450s with respect to CFP was further exacerbated when both alleles were combined (χ^2 = 38.6; p < 0.0001). Double homozygote susceptible (SS/SS) mosquitoes had highest chance of surviving CFP exposure (OR = 5.7; p < 0.0001) compared with their double homozygote pyrethroid-resistant (RR/RR) counterparts (Figure 2F). Also, double heterozygote (RS/RS) mosquitoes had more chance of survival than RR/RR (OR = 4.2; p < 0.0001) (Figure 2F), with RS/RR also having higher tolerance compared with the RR/RR individuals (OR = 0.1; p < 0.0001). All these data confirmed that possessing pyrethroid-resistant allele for *CYP6P9a/-b* increases the insecticidal potency of CFP.

Impact of CYP6P9a/-b_R on the efficacy of chlorfenapyr-based nets compared to pyrethroid only

Tunnel tests were performed for IG2 and Royal Guard nets using field *An. funestus* mosquitoes from Malawi, and similar tests were performed against pyrethroid-only net Interceptor (n = 215), CFP-based net (100 mg/m^2, n = 108 and 200 mg/m^2, n = 73), and the dual LLINs IG2 (n = 232) on the hybrid strain FANG/FUMOZ. In addition, around 1,400 mosquitoes (267 in the control hut, 312 in the hut with Interceptor, 322 in the hut with IG2, and 501 in the hut with CFP-100 net) from the hybrid strain were released and recaptured in EHT for 1 week to establish the impact of CYP6P9a/b on the efficacy of these tools in semi-field conditions.

Tunnel assay results

High mortality rates were observed in the hybrid FUMOZ/FANG strain against the CFP-only nets (100 mg/m^2, 57.9%, confidence interval [CI] = 48.2–67.8 and 200 mg/m^2, 77.2%, CI = 68.9–85.4) (Figure S2A). The dual AI net IG2 also induced a significantly higher mortality (80.6%) compared to pyrethroid-only net Interceptor (52.4%) (χ^2 = 27.6; p < 0.0001) and control (25.2%) (χ^2 = 244.07; p < 0.0001) (Figure S2A). The penetration rate was significantly higher in 100 mg/m^2 CFP-only net in tunnel tests compared to all other nets (IG2 and Interceptor; p < 0.0001) and consequently with higher blood-feeding rate (p < 0.0001) (Figure S2A). The *An. funestus* population from Malawi displayed a mortality rate of 44.93% for IG2 and 40.83% for Royal Guard in tunnel test (Figure S2B), and no blood feeding was recorded in these field mosquitoes for all the nets tested. Genotyping of *An. funestus* from Malawi after exposure to Interceptor G2 in tunnel revealed very high frequency (>95%) of both *CYP6P9a* and *CYP6P9b* in dead and alive, blood-fed and unfed mosquitoes (Figures S3A–S3D) preventing us from establishing the association between these markers and the efficacy of the CFP-based net IG2. Genotyping of these

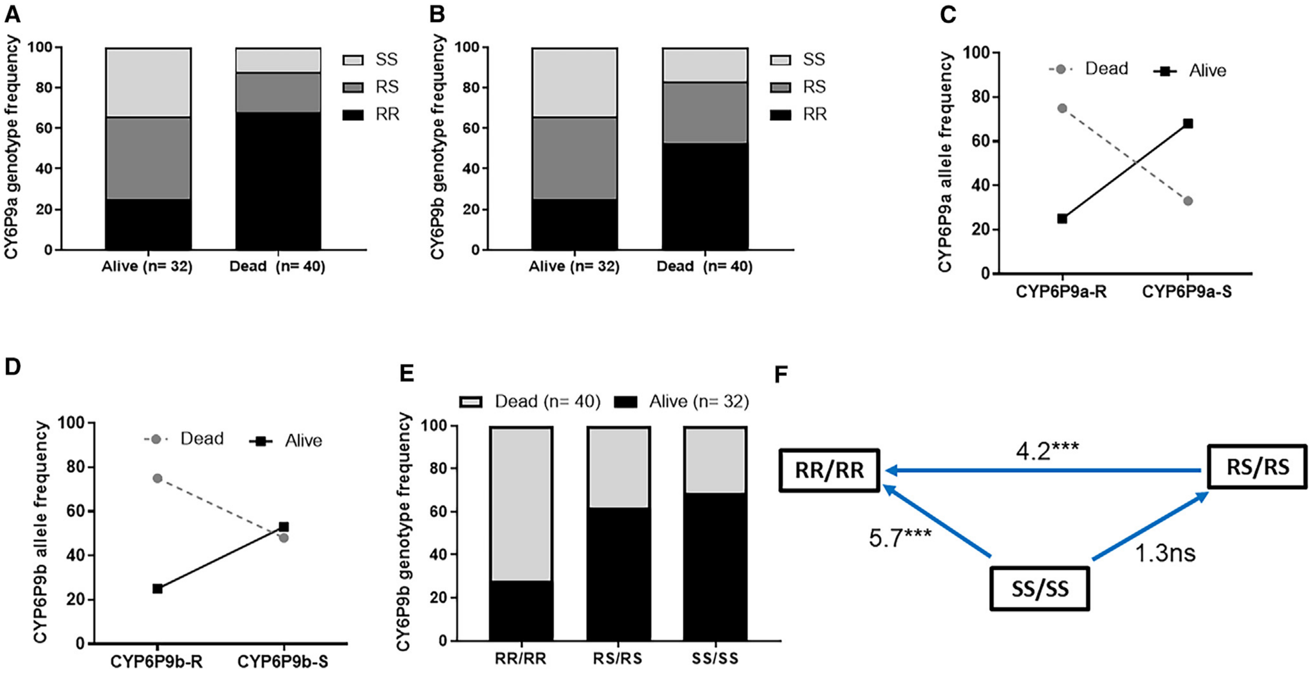

Figure 2. Impact of *CYP6P9a/-b* on the efficacy of chlorfenapyr using CDC bottle assays

(A–F) Distribution of the *CYP6P9a* genotypes (A) and alleles (B) between alive and dead mosquitoes after exposure to CFP; distribution of the *CYP6P9b* genotypes (C) and alleles (D) between alive and dead mosquitoes after exposure to CFP; distribution of the combined genotypes at the *CYP6P9a_R* and *CYP6P9b_R* alleles between alive and dead mosquitoes after exposure to CFP (E); and odds ratio calculations comparing the ability of double homozygote resistant mosquitoes to survive CFP exposure to other genotype combinations (F). *n* = total number of mosquitoes from each phenotype that were successfully genotyped. Fisher test was used to discern significant differences.

markers in the FG/FZ strain revealed a highly significant difference in the distribution of genotypes between the dead and alive mosquitoes for all the nets ($\chi^2 > 20$; $p < 0.0001$) (Figure S4). Analysis of the correlation between genotypes and mortality revealed that *CYP6P9a* homozygous resistant mosquitoes (RR) exhibited higher survival rate on exposure to the pyrethroid-only net Interceptor (Figure S4A) compared to homozygote susceptible mosquitoes (SS) (OR = 123; CI = 18–1,273; $p < 0.0001$) (Table S4). Also, heterozygote mosquitoes (RS) had higher survival rate from exposure to this net compared to homozygote susceptible ones (OR = 66; CI = 10–684; $p < 0.0001$) (Table S4). However, mosquitoes with homozygote-resistant genotype (RR) had less chance of surviving CFP-only net (CFP-100 net) than heterozygotes (OR = 0.3; CI = 0.1–0.8; $p = 0.02$) and homozygote susceptible (OR = 0.8; CI = 0.3–1.9; $p = 0.8$) indicating the high insecticidal potency of CFP on pyrethroid-resistant mosquitoes. No difference was observed for the dual AI interceptor G2 (Table S4), suggesting a balancing effect between pyrethroid and CFP. Contrary to *CYP6P9a*, *CYP6P9b* had less pronounced impact on the CFP-based nets (Figure S5A), with analysis of the correlation between genotypes and mortality revealing no difference between RR and SS (OR = 0.8; CI = 0.3–1.9; $p = 0.8$) for *CYP6P9b*.

The blood-feeding rate was significantly lower in tunnels with treated nets (Interceptor = 40.3%, IG2 = 31.3%, CFP-100 = 57.9%, and CFP-200 = 2.4%) compared to the untreated (73.1%) ($p < 0.05$), and the blood-feeding success of CFP-200 was significantly lower compared to all other nets ($p < 0.0001$) (Figure S3A). Genotyping of blood-fed and unfed mosquitoes revealed that homozygote-resistant mosquitoes (bearing *CYP6P9a_R* and *CYP6P9b_R* markers) were significantly more likely to blood feed than RS (OR = 4.5; $p < 0.001$) in the presence of Interceptor (Table S4). Also, RS ones were more able to blood feed in the presence of this net compared to SS mosquitoes (OR = 2.0; $p < 0.001$). The mosquitoes harboring the resistant allele were also more able to blood feed in tunnel with Interceptor (Figures S4D and S5D) and IG2 net (Figures S4E and S5E) than those with the wild-type allele, showing that in addition to increased survival, *CYP6P9a/-b* confer blood feeding advantage in the presence of pyrethroid-based nets (Table S4). However, no significant difference was observed in the blood-feeding ability of RR (for *CYP6P9a*) compared to RS or SS mosquitoes for the CFP-only net CFP-100 (Figure S4F; Table S4) despite the slight trend observed for *CYP6P9b* (Figure S5F; Table S4).

Experimental hut trial results

Similar patterns in mortality rates were observed for the hybrid strain using EHT, with all the CFP-based nets inducing significantly higher mortality ($p < 0.0001$) than pyrethroid-only nets (Figure S2C). A mortality rate of 92.6% (CI = 90.3–94.9) was obtained for CFP-only net (100 mg/m^2), 83.4% (CI = 79.3–87.5) for IG2, and 66.5% (CI = 61.3–71.7) for the pyrethroid-only net Interceptor (Figure S2C). Because genotyping of *CYP6P9a_R/b_R* markers using mosquitoes from Malawi revealed a very high frequency of the mutant allele close to fixation, the impact of

CellPress
OPEN ACCESS

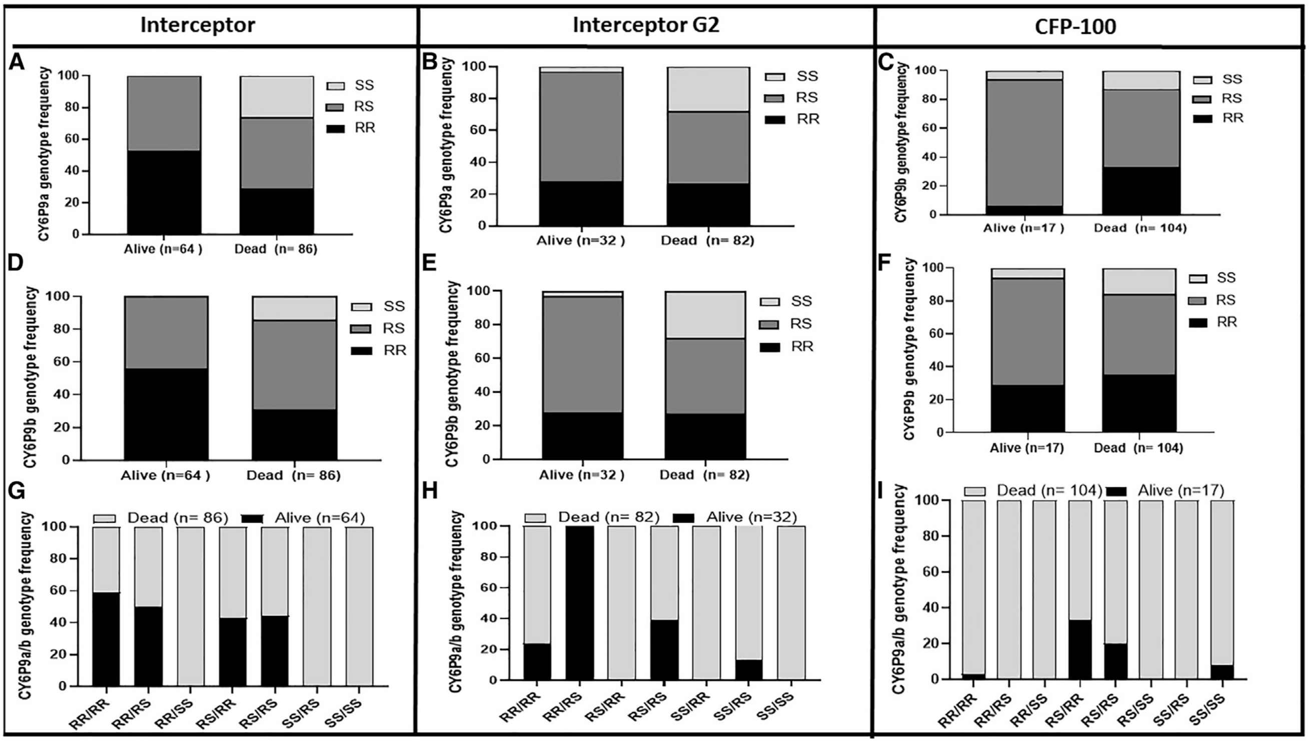

Figure 3. Impact of the *CYP6P9a/-b* on the efficacy of CFP-based nets on *An. funestus* in EHT

(A–I) *CYP6P9a* genotype distribution between alive and dead mosquitoes after exposure to Interceptor (A), IG2 (B), and CFP-100 (C); *CYP6P9b* genotype distribution between alive and dead mosquitoes after exposure to Interceptor (D), IG2 (E), and CFP-100 (F); and combined *CYP6P9a* and *CYP6P9b* genotype distribution between alive and dead mosquitoes after exposure to Interceptor (G), IG2 (H), and CFP-100 (I). For genotype: RR, homozygote resistant; RS, heterozygote; and SS, homozygote susceptible. *n* = total number from each phenotype that were successfully genotyped. Fisher test was used to discern significant differences.

CYP6P9a/b on the efficacy of CFP-based control tools was only performed using the F_3 of the FG/FZ hybrid strain in EHT. A total of 385 mosquitoes were successfully genotyped for Interceptor net (n = 150), IG2 (n = 114), and CFP-100 (n =121), revealing a highly significant difference in the distribution of genotypes between the dead and alive mosquitoes for all the nets ($\chi^2 > 20$; $p < 0.0001$) in EHT (Figure 3). *CYP6P9a* homozygous resistant mosquitoes (RR) exhibited higher survival rate on exposure to the Interceptor net (Figure 3A) compared to homozygote susceptible mosquitoes (SS) (OR = 47.5; CI = 7.6–497.8; $p <$ 0.0001) (Table S5). Also, heterozygote mosquitoes (RS) had higher survival rate from exposure to this net compared to homozygote susceptible (OR = 27.1; CI = 4.5–285.4; $p < 0.0001$) (Table S5). However, mosquitoes with homozygote-resistant genotype (RR) had less chance of surviving CFP-only (CFP-100 net) than heterozygotes (OR = 0.1; CI = 0.04–0.2; $p < 0.0001$) and homozygote susceptible (OR = 0.3; CI = 0.3–1.1; $p = 0.1$), confirming the high insecticidal potency of CFP on pyrethroid-resistant mosquitoes. As observed with *CYP6P9a*, a similar pattern was noticed for *CYP6P9b* (Figures 3B andS5A), with analysis of the correlation between genotypes and mortality revealing that *CYP6P9b* homozygous resistant mosquitoes (RR) had higher chances to survive exposure to the Interceptor compared to homozygote susceptible mosquitoes (OR = 47.5; CI = 7.6–497.8; $p < 0.0001$) (Table S5). For the dual AI net IG2, having the resistant allele did not significantly confer increased survival advantage (OR = 1.7; CI = 1–3.06; $p = 0.06$) as observed in tunnel tests, although here, RR and RS individuals had a higher chance of surviving than their SS (RR vs. SS: OR = 13.2; CI = 2.2–44.3; $p = 0.003$, RS vs. SS: OR = 13.2; CI = 2.2–44.3; $p = 0.003$) counterparts (Table S5).

In EHT, the blood-feeding rate was significantly lower in treated huts (Interceptor = 15.4%, IG2 = 10.6%, and CFP-100 = 14.6%) compared to the hut with untreated net (68.5%) ($p < 0.0001$). However, blood-feeding success did not significantly differ between treatments ($p > 0.05$) (Figure S2C). CFP-100 net had a higher blood-feeding rate compared to other treatments (Figure S2C). Genotyping revealed that homozygote-resistant mosquitoes (bearing *CYP6P9a_R* and *CYP6P9b_R* markers) were significantly more likely to blood feed than RS (OR = 3.7; $p < 0.0001$) and SS (OR = 5.1; $p < 0.001$) when exposed to Interceptor (Table S5). The mosquitoes harboring the resistant allele were more able to blood feed in Interceptor-treated hut than those with the wild-type allele (OR = 2.7; CI = 0.3–1.7; p = 0.001) confirming that in addition to increased survival, the *CYP6P9a/b* confer blood-feeding advantage in the presence of a pyrethroid-only net (Table S5). A similar trend was observed for IG2, but no difference was observed in the blood-feeding ability of RR compared to RS or SS mosquitoes for the CFP-based nets CFP-100 (Figures S6 and S7).

The exophily rate in the hut with untreated net (10.10%) was significantly lower than that in the IG2 hut (27%) ($p < 0.0001$), CFP-100 hut (18%; $p = 0.003$), and Interceptor one (33%; $p < 0.0001$) (Figure S2C). Genotyping results revealed that homozygote-resistant mosquitoes (RR) for both *CYP6P9a* (Figure S6) and *CY6P9b* (Figure S7) had a greater ability to stay in the room with Interceptor compared to RS (OR = 1.7; p =0.08) and SS ones (OR = 0.3; $p = 0.004$), which tended mainly to exit from the treated huts. The same trend was observed for the IG2 (Figures S6 and S7), but no difference was observed for the CFP-100 net (Figures S6 and S7) as this insecticide does not have any repellency effect. At the allelic level, no difference was observed between R and S for all the nets.

Combined impact of *CYP6P9a* and *CYP6Pb* on the efficacy of chlorfenapyr-based nets in experimental hut trial

We also assessed how combinations of genotypes at both genes impact the efficacy of a CFP-based net, focusing mainly on mortality and blood feeding. Analysis of the impact of combined genotypes on mortality/blood feeding after exposure to Interceptor, IG2, or CFP-100 net confirmed the independent segregation of genotypes at both genes with several combinations of genotypes observed, including RR/RR, RR/RS, RS/RS, RS/SS, and SS/SS (Figure 3). Comparison of the distribution of combined genotypes revealed that double homozygote resistant (RR/RR) mosquitoes at both loci had a greater ability to survive exposure to Interceptor than most of the other combinations (Figure 3G), particularly when compared to double susceptible mosquitoes (SS/SS) (OR = 25.04; CI = 4.1–271.2; $p < 0.0001$). This genotype combination had less impact on interceptor G2 (Figure 3H), whereas a strong negative association was observed for CFP-100 net (Figure 3I), particularly in RR/RR vs. RS/RS comparison (OR = 0.1; CI = 0.05–0.3; $p < 0.0001$). This negative association was even stronger in tunnel assay where analysis of the combined genotype distribution for blood feeding also revealed a significantly increased ability of RR/RR mosquitoes to blood feed in the presence of Interceptor (OR = 3.2; CI = 1.2–7.6; $p = 0.01$) or IG2 (OR = 5.9; CI = 1.6–17.4; $p = 0.002$) compared to SS/SS mosquitoes (Table S7), but the difference was not significant for CFP-only net (OR = 2.5; CI = 0.8–7.01; $p = 0.01$). In EHT, no difference was observed in the blood feeding of resistant mosquitoes compared to the susceptible ones for all the nets (Table S8).

Association between CYP6P9a/b markers and clothianidin resistance after CDC bottle assays

As the frequency of the resistant allele was very high in Malawi *An. funestus* (*CYP6P9a_R* = 73% and *CYP6P9b_R* = 99%), no significant differences ($p = 0.4$) were obtained in the distribution of genotypes between dead and alive mosquitoes following exposure to neonicotinoids (Figure S2). However, homozygote-resistant mosquitoes for *CYP6P9a* were predominantly alive compared to the dead ones (OR = 1.1; CI = 0.7–2.8; $p = 0.3$), indicating that the resistant allele for this gene could confer survival advantage, though not significant. To better establish the impact of these markers on neonicotinoid resistance, the hybrid FG/FZ mosquitoes were used for further analysis. In this strain, the *CYP6P9a* homozygous resistant mosquitoes (RR) and heterozygous ones (RS) were significantly more able to survive clothianidin exposure than homozygote susceptible mosquitoes (Figure 4A). A strong association between the *CYP6P9a* resistance allele and the ability to survive CLTD exposure was noted for RR vs. SS (OR = 7.5; $p = 0.001$) and RS vs. SS (OR = 3.5; $p = 0.02$) (Table S2). This was confirmed at the allelic level (OR = 2.2; $p < 0.005$ for R vs. S) (Table S2; Figure 4C). For *CYP6P9b*, homozygous resistant mosquitoes (RR) and heterozygous ones (RS) were also significantly more able to survive CLTD exposure than the homozygous susceptible mosquitoes (SS): RR vs. SS (OR = 7.08; $p = 0.002$) and RS vs. SS (OR = 3; $p = 0.05$) (Table S2; Figure 4B). When combining both markers, double homozygote resistant individuals (RR/RR) had more chance to survive clothianidin exposure compared to all other genotypes (Figures 4E and 4F).

Impact of CYP6P9a_R/-b_R markers on clothianidin-based IRS interventions in experimental hut trials

Figure 2D summarizes the results of the efficacy of IRS products in experimental huts using the FG/FZ hybrid strain. Compared to the untreated hut, all experimental huts induced significantly higher mortality ($p < 0.0001$). The mortality was significantly higher in clothianidin- and Fludora Fusion-treated huts (72.5% and 79.6%, respectively) compared to deltamethrin-sprayed huts (43.8%). Also, a significant reduction in the blood-feeding rate was observed in the treated huts compared to the untreated hut ($p < 0.05$), which was more pronounced in the case of huts sprayed with Fludora Fusion ($p < 0.001$). Although the induced exophily was significantly elevated ($p < 0.05$) in the treated huts compared to the untreated one, no difference was observed between treated huts (Table S4).

After genotyping, a significant association was found between possession of the resistant allele for both genes and the ability of mosquitoes to survive exposure to deltamethrin indoor spray ($\chi^2 = 21.3$; $p < 0.0001$) (Figure 5). Comparison of allele frequencies showed that possessing the resistant allele increased the chance of surviving exposure to deltamethrin more than 3-fold (Table S6). Less impact was observed for clothianidin and Fludora Fusion where comparison of genotypic and allelic frequencies revealed that possessing CYP6P9a_R/-b_R did not increase significantly the ability to survive exposure to clothianidin ($\chi^2 = 1.3$; $p = 0.5$) and Fludora Fusion ($\chi^2 = 1.02$; $p = 0.06$) (Figures 5B, 5C, 5E, and 5F; Table S6). This shows that although CYP6P9a/b-resistant mosquitoes are more able to withstand deltamethrin and clothianidin exposure in CDC bottle assay, they remain susceptible to the new IRS formulation Fludora Fusion in the EHT.

A significant association was observed between possession of the resistance alleles (CYP6P9a/b_R) and the ability to take blood meals in the presence of deltamethrin ($\chi^2 = 21.7$; $p < 0.0001$) (Figures S8and S9) but not Fludora Fusion and clothianidin (Table S6).

Assessing the impact of *CYP6P9a/-b* on clothianidin and chlorfenapyr resistance using transgenic *Drosophila* flies

Bioassays performed with 50 μg/mL clothianidin and 10 μg/mL of CFP revealed that flies expressing CYP6P9a and CYP6P9b

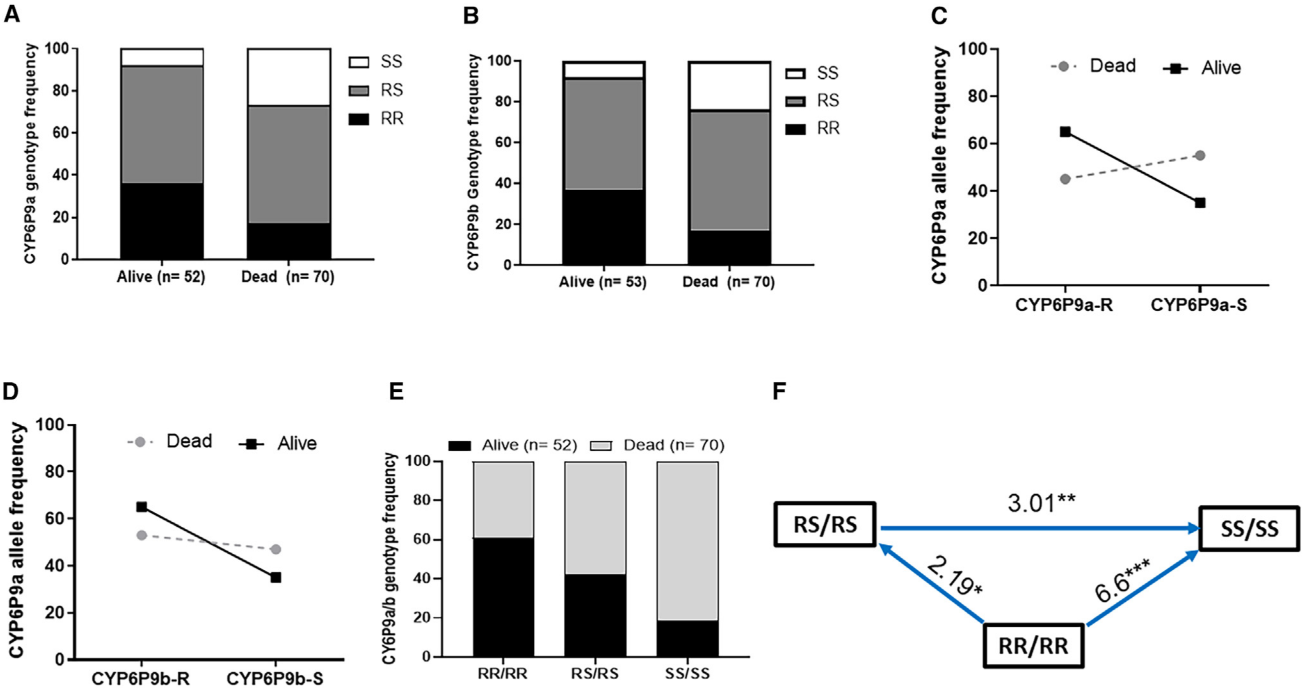

Figure 4. Impact of the duplicated *CYP6P9a/b* on the efficacy of clothianidin on *An. funestus* in CDC bottle assays

(A and B) Distribution of the *CYP6P9a* genotypes and alleles between alive and dead mosquitoes after exposure to CLTD.

(C and D) Distribution of the *CYP6P9b* genotypes and alleles between alive and dead mosquitoes after exposure to CLTD.

(E) Distribution of the combined genotypes at the *CYP6P9a* and *CYP6P9b* loci between alive and dead mosquitoes after exposure to CLTD.

(F) Odds ratio calculations comparing the ability of double homozygote resistant mosquitoes to survive CLTD exposure to other genotype combinations. *n* = total number from each phenotype that were successfully genotyped. Fisher test was used to discern significant differences.

were significantly more resistant to clothianidin 12 h post exposure than the control flies, with average mortalities at 12 h of 45.05% ± 7.03% for CYP6P9a ($p < 0.001$) and 30.1% ± 2.9% for CYP6P9b ($p < 0.001$) compared to the control flies (73.9% ± 3.3%) (Figure 6A). However, no differences were obtained between these transgenic flies and control at 24-h exposure (Figure 6A). In contrast experimental flies overexpressing these two P450s were more susceptible to CFP at 6 h, 12 h, and 24 h post exposure compared to control flies, with average mortalities at 12 h of 62.3% ± 4.1% for CYP6P9a ($p < 0.001$) and 61.1% ± 5.9% for CYP6P9b ($p < 0.001$) compared to the control (37.6% ± 5.6%) (Figure 6B). This indicates that the overexpression of these P450s alone can confer clothianidin resistance while increasing the susceptibility to CFP.

Assessing the impact of *CYP6P9a/-b* on clothianidin and chlorfenapyr resistance after gene knockdown through RNAi

CYP6P9a knockdown using RNAi confirmed the above phenotypes in *An. funestus* mosquitoes, with mortalities increasing significantly in mosquitoes injected with ds*CYP6P9a* compared to mosquitoes injected with ds-GFP and non-injected mosquitoes (Figure 6C). In the ds-*CYP6P9a*-injected mosquitoes, mortality rates were 51.5% ± 3.4%; 78.4% ± 9.1%, and 94.7% ± 7.8% at 24 h, 48 h, and day 3 post exposure to clothianidin and then 100% after day 3 (Figure 6C). In the ds-GFP, the mortality was significantly lower ($p < 0.0001$) varying from 35.9% ± 4.9% at 24 h to 95.1% ± 4.22% at day 6 and then 100% at day 7 post exposure. A similar trend was observed in non-injected mosquitoes where a mortality rate of 35.9% ± 4.9% was obtained at 24 h, 89.3% ± 8.9% at day 6, and then 100% at day 7 post exposure. The opposite pattern was seen for CFP where the mortality in ds*CYP6P9a* was significantly lower than in the ds-GFP-injected and non-injected mosquitoes (Figure 6D). A mortality rate of 11% ± 3.4% was recorded at 24 h post exposure to CFP in ds-*CYP6P9a*-injected mosquitoes compared to 22.5% ± 4.9% for ds-GFP-injected ($p < 0.05$) and 21.05% ± 2.3% for non-injected mosquitoes ($p < 0.05$). At day 7 post exposure, the mortality of 38% ± 4.4% was recorded in ds*CYP6P9a*-injected mosquitoes compared to 77.7% ± 6.3% for ds-GFP-injected ($p < 0.0001$) and 95.4% ± 4.6% for non-injected mosquitoes ($p < 0.0001$). Unfortunately, due to the low number of mosquitoes, RNAi assay was performed only for CYP6P9a.

In vitro validation of clothianidin and chlorfenapyr metabolism using recombinant *CYP6P9a*

Substrate depletion assays with the recombinant *An. funestus CYP6P9a* revealed moderate metabolism of both clothianidin and CFP, with 26.5% ± 4.8% clothianidin depleted ($p < 0.05$ versus $-NADP^+$ incubation) and 34.09% ± 3.08% CFP depleted ($p < 0.02$ vs. $-NADP^+$ incubation) following incubation for 90 min (Figure 6E). Further, metabolism of CFP was confirmed from the appearance of tralopyril (a bioactivated product of

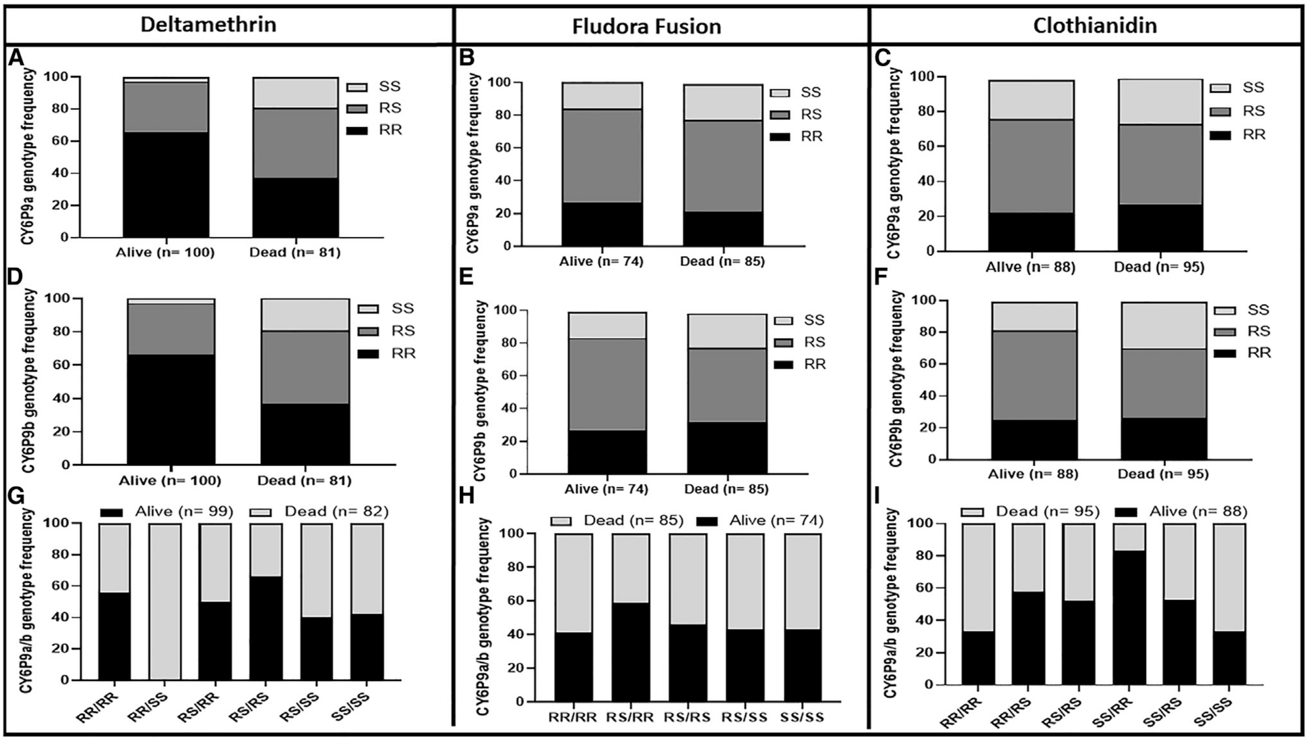

Figure 5. Impact of *CYP6P9a*/*-b* on the efficacy of CFP-based nets on *An. funestus* in EHT

(A–F) *CYP6P9a* genotype distribution between alive and dead mosquitoes after exposure to deltamethrin (A), Fludora Fusion (B), and clothianidin (C) and *CYP6P9b* genotype distribution between alive and dead mosquitoes after exposure to deltamethrin (D), Fludora Fusion (E), and clothianidin (F). For genotype: RR, homozygote resistant; RS, heterozygote; and SS, homozygote susceptible. *n* = total number from each phenotype that were successfully genotyped. Fisher test was used to discern significant differences.

N-dealkylation of the ethoxymethyl group of CFP), which eluted around the 14th minute in the $+NADP^{+}$ incubation samples (Figure 6F).

DISCUSSION

Using DNA-based markers and extensive functional analyses, this study established that the duplicated cytochrome P450 genes, *CYP6P9a* and *CYP6P9b*, known as proficient pyrethroid metabolizers, are driving cross-resistance to clothianidin but, in contrast, boost the efficacy of CFP.

The pyrethroid-resistant *An. funestus* strains exhibit a greater susceptibility to chlorfenapyr than neonicotinoids

Both *An. funestus* populations from Malawi and the crosses were susceptible to CFP (100 μg AI/bottle). This susceptibility of *An. funestus* to CFP has been previously reported in many other countries,[32] supporting the choice of this insecticide for new vector control tools. Large-scale susceptibility testing of this insecticide with 100 μg AI/bottle as done in this study revealed susceptibility against malaria vector populations from about 16 countries, including mosquitoes with multiple resistance mechanisms to pyrethroids.[33] This high susceptibility to CFP is mainly due to the fact that it is a pro-insecticide that becomes toxic when the N-ethoxy methyl group is removed through P450-mediated oxidation creating the toxic metabolite tralopyril (CL303268). The resulting tralopyril disrupts the proton gradient across the mitochondrial membranes and impairs the production of ATP (oxidative phosphorylation),[34,35] leading to cell death. This mode of action of CFP is completely different from standard neurotoxic insecticides such as DDT and pyrethroids, etc., assuming less chance for cross-resistance, although some *An. gambiae* populations from the Agréby-Tiassa region of southeast Côte d'Ivoire and agricultural settings from Cameroun, Ghana, and DRC presented a reduced susceptibility to the DD of CFP.[32,36,37] In contrast to CFP, neonicotinoids induced low mortality in *An. funestus* populations from Malawi and the hybrid strain FANG/FUMOZ when using absolute ethanol/acetone alone as solvent, although the addition of MERO significantly increases the efficacy. The 24-h to 7-day post-exposure mortality has revealed in general low mortality of mosquitoes in these strains against the three neonicotinoids tested (clothianidin, imidacloprid, and acetamiprid) when these insecticides were diluted in acetone/ethanol alone, with a slight increased mortality from day 1 to day 7. This confirms the slow-acting effect of neonicotinoids as previously reported by several studies[38,39] and indicates that the addition of MERO to acetone/ethanol while maximizing the efficacy of clothianidin can also prevent early detection of resistance. In fact, the addition of MERO (89 PPM) to acetone significantly increased the efficacy of neonicotinoids on the mosquitoes tested, with 100% mortality observed 24 h post exposure for both strains. As previously reported, the high

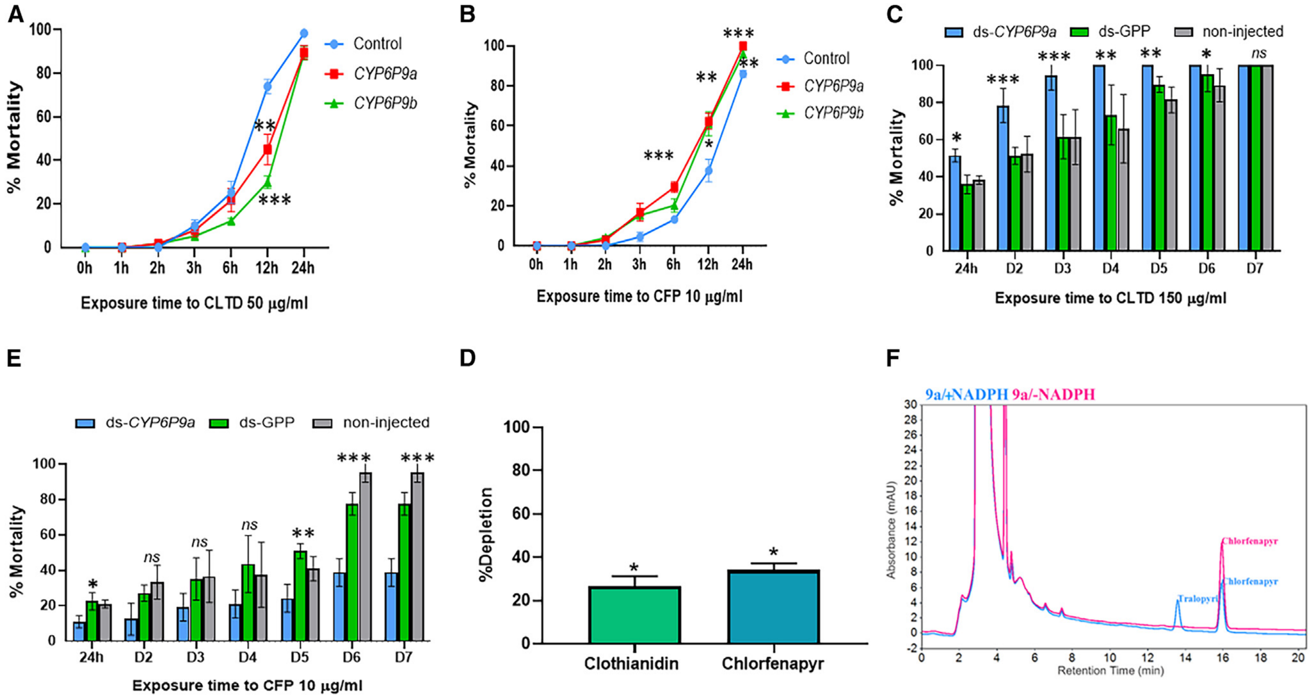

Figure 6. ***In vivo*** **and** ***in vitro*** **functional validation of the role of CYP6P9a/b in clothianidin and chlorfenapyr resistance**

(A–F) Mortality pattern of GAL4 x UAS-CYP6Pa and GAL4 x UAS-*CYP6P9b* transgenic flies exposed to clothianidin (A) and chlorfenapyr (B); mortality of the hybrid FANG/FUMOZ after RNAi knocked down each of the duplicated CYP6P9a genes 7 days post exposure to clothianidin (C) and chlorfenapyr (D); and percentage depletion (mean ± standard deviation [SD]) of clothianidin and chlorfenapyr at 90 min (E) with formation of tralopyril as the primary product of bioactivation of chlorfenapyr (F). Asterisks indicate the difference between each ds-P450 gene in comparison to ds-GFP control and un-injected mosquitoes (**p* < 0.05, ***p* < 0.01, ****p* < 0.001). In this figure, values represent the mean of 4–5 biological replicates, and error bars represent ±SD of mean. χ^2 test was used to discern significant differences.

mortality observed when using acetone + MERO as a solvent could be explained by the properties of MERO to increase the solubility of neonicotinoids,[40] preventing the crystallization.[41,42] Such high efficacy induced by acetone + MERO as solvent confirms that this is a suitable solvent for neonicotinoids, as previously reported by Tchouakui and collaborators and the recent WHO manual for monitoring insecticide resistance in mosquito vectors.[42,43] However, ethanol or acetone alone is still very useful in capturing variability between populations and establishing the impact of some markers on mosquitoes' ability to withstand exposure to these insecticides. Moreover, pre-exposure to PBO significantly restored the susceptibility to the three neonicotinoids (when using acetone alone as solvent), indicating the implication of P450s to the reduced susceptibility observed.

Chlorfenapyr-based tools are turning mosquito molecular defense against them, explaining their reported efficacy

To combat the increasing insecticide resistance in mosquitoes, new insecticide molecules and combinatorial strategies have now been adopted. In this study, the dual AI net IG2 combining CFP and alpha-cypermethrin and the CFP-only net induced significantly higher mortality on the strains tested both in tunnel tests and EHTs compared to the pyrethroid-only net. In the hybrid strain, FANG/FUMOZ, a mortality rate of 80.6% was obtained for IG2 and 77.2% for the CFP-only net (200 mg) compared to the pyrethroid-only net Interceptor where only 52.4% mortality was obtained. The low mortality response obtained with the pyrethroid-only net could be associated to pyrethroid resistance driven by *CYP6P9a/b* as previously reported in this strain[27,28] and the *An. funestus* from Malawi.[31] The greater efficacy of the CFP-based net observed in this study as previously reported in Benin, Tanzania, and Cameroon[24,44,45] should be therefore attributed to the potential bioactivation of CFP by P450s in pyrethroid-resistant mosquitoes. IG2 could therefore be an efficient replacement for pyrethroid-only ITNs for the reduction of malaria transmission malaria vectors as observed in Benin and Tanzania.[19,46] In Tanzania, RCTs highlight that *An. funestus* was the dominant vector, where previous work showed that CYP6p9a/-b were the main drivers of pyrethroid resistance.[26] This explains the high efficacy of IG2 on the strain tested. For Benin, the predominant vector was rather *An. gambiae*, and probably the same effect as for CYP6P9a/b is observed, but more work is needed to validate the role of the main pyrethroid resistance genes on susceptibility to CFP.

Cytochrome P450 substrate promiscuity is a double-edged sword for the success of vector control

After CDC bottle assays, mosquitoes harboring the CY6P9a/b-resistant allele had a higher ability to survive CLTD exposure.

The association between CY6P9a/b-resistant allele and resistance to clothianidin could be associated with the metabolism of neonicotinoids by *CY6P9a/b* as previously shown for carbamates. A recent study by Mugenzi et al.[47] revealed after RNA sequencing that cytochrome P450s, notably the duplicated *CYP6P9a* and *-b* in Southern Africa, are playing a major role in carbamate resistance in *An. funestus*. They demonstrated through *in vivo* and *in vitro* functional analyses that *CYP6P9a* and *-b* metabolize carbamates and that their overexpression is sufficient to confer resistance to this insecticide class similar to the pyrethroids. In this study, we notice that *CYP6P9a* also metabolizes clothianidin at a higher rate than carbamate. This is the first study showing a cross-resistance between pyrethroid-resistant genes and neonicotinoid resistance. This was confirmed by the transgenic flies' assays and RNAi where mosquitoes/flies expressing the *CYP6P9a*-resistant genes had higher survival ability against clothianidin. Our results highlight the potential danger that complex evolution of resistance to an insecticide class intensely used in the field such as pyrethroids could pose to the efficacy of new insecticides. Such cross-resistance issues should be taken into account to implement robust insecticide-based intervention using molecular tools available for informed decision-making.

Interestingly, we observed in this study for the first time a negative association between these pyrethroid resistance markers and CFP resistance. Mosquitoes harboring the resistant allele for both *CYP6P9a* and *CYP6P9b* markers were less tolerant to CFP in CDC bottle assay, tunnel tests, and EHTs. This was further confirmed by the knockdown of these genes in mosquitoes as well as the transgenic flies. Furthermore, heterologous analysis of CFP metabolism indicates that the principal metabolite produced by these *CYP6P9a* was tralopyril, the N-dealkylated insecticidal form that disrupts oxidative phosphorylation. This is the first study establishing such negative association between pyrethroid-resistance markers and CFP resistance, although *CYP6P3*, *CYPJ5*, and *CYP9K1* in *An. gambiae* and *CYP9J32* in *Ae. aegypti* were recently shown to bio-activate the CFP[30] as noticed here with *CYP6P9a*. Previous studies have also indicated that CFP is more toxic to pyrethroid-resistant pests including cattle horn fly or the tobacco budworm where resistance to pyrethroid is driven by P450 overexpression.[48,49] All this shows that pyrethroid-resistant populations of *An. funestus* mosquitoes where CYP6P9a/-b are overexpressed may have an enhanced capacity to activate CFP, resulting therefore in improved susceptibility to a novel WHO-recommended insecticide. However, caution must be applied in correlating metabolic pyrethroid resistance with CFP activation as recent *in vitro* metabolism assay revealed that only four of the nine P450s tested were capable of metabolizing CFP, and rates of metabolism differed widely.[30] By showing negative cross-resistance with CFP, it goes further by allowing a control program to take advantage of this in the context of such a phenomenon based on evidence from the field before implementing a strategy where CFP-based tools could be promoted. Therefore, similar work with other markers is needed to show the extent of such negative cross-resistance in line with contrasting patterns of resistance as seen in *An. funestus* where contrasting resistance fronts are present with different resistance genes driving it, for example, the *CYP9K1* predominant in east Africa,[50] *CYP6P4* in the west,[27] and *CYP325A* and 4.3kb-SV in central Africa.[27] Similar work should be also extended to other major vectors such as gambiae, etc. This is also an advantage of such markers over relying on whole-genome sequencing as they provide easy use to tackle key questions with field samples.

Conclusion

This study using recently detected DNA-based markers of P450-linked pyrethroid resistance to directly establish how pyrethroid-resistant mosquitoes interact with novel insecticides revealed that the duplicated CYP6P9a/b pyrethroid-resistance genes are driving cross-resistance to clothianidin but, in contrast, boost the efficacy of CFP. This highlights the risk that pyrethroid resistance escalation poses to the efficacy of other classes of insecticides such as neonicotinoids but at the same time reveals that CFP-based control interventions such as IG2, which is currently largely distributed across Africa for malaria control, could be very efficient against some P450-based pyrethroid-resistant mosquitoes.

Limitations of the study

One of the major limitations of this study is the lack of metabolite identifications using mass spectrometry. Specifically, tralopyril serves as the diagnostic metabolite for CFP metabolism. In the case of clothianidin, mass spectrometry analysis could have been carried out to identify its metabolites. Additionally, insecticide metabolites generated by the transgenic *Drosophila* flies could have been determined using needle biopsy spray mass spectrometry. These important studies will be the subject of future work.

STAR★METHODS

Detailed methods are provided in the online version of this paper and include the following:

- KEY RESOURCES TABLE
- RESOURCE AVAILABILITY
  - Lead contact
  - Materials availability
  - Data and code availability
- EXPERIMENTAL MODEL AND STUDY PARTICIPANT DETAILS
  - Trials design
  - *Anopheles funestus* strain used
  - Ethical approval
- METHOD DETAILS
  - Assessing the impact of *CYP6P9a_R*/*-b_R* on the efficacy of chlorfenapyr and clothianidin using CDC bottle assay
  - Assessing the impact of *CYP6P9a_R*/*-b_R* on the efficacy of chlorfenapyr-based nets using tunnel tests
  - Assessing the impact of *CYP6P9a_R*/*-b_R* on the efficacy of CFP-based nets using experimental hut trials
  - Assessing the impact of *CYP6P9a_R*/*-b_R* on the efficacy of clothianidin-based IRS using experimental hut trials
  - Assessing the impact of CYP6P9a/-b on efficacy of chlorfenapyr and clothianidin using *in vivo* transgenic expression in with *Drosophila melanogaster*
  - Assessing the impact of CY6P9a/-b on efficacy of chlorfenapyr and clothianidin using RNA-interference
  - Investigating chlorfenapyr and clothianidin metabolising activity of *CYP6P9a* using *in vitro* protein expression and metabolism assays

- QUANTIFICATION AND STATISTICAL ANALYSIS
  - Experimental hut trial
  - Test of association between the P450 genes (*CYP6P9a*/-*b*) and the entomological outcomes

SUPPLEMENTAL INFORMATION

Supplemental information can be found online at https://doi.org/10.1016/j.celrep.2024.114566.

ACKNOWLEDGMENTS

We gratefully acknowledge the contribution of Innovative Vector Control Consortium (IVCC) and Badische Anilin- & Soda-Fabrik (BASF), who supported and provided the insecticides and nets for this study, and to the population of Elende for their collaboration in all EHTs.

This work was supported by the Wellcome Trust Senior Research Fellowship in Biomedical Sciences (217188/Z/19/Z to C.S.W.) and Bill and Melinda Gates Foundation Investment (ID INV-006003 to C.S.W).

AUTHOR CONTRIBUTIONS

Conceptualization: C.S.W. and M.T. Investigation: M.T., R.F.T., T.A., S.L.N.-Y., B.D.M., T.M., M.K.M., S.S.I., A.M., and L.M.J.M. Data curation and analysis: C.S.W. and M.T. Funding acquisition: C.S.W. Writing – original draft: M.T. Writing – review & editing: M.T., S.S.I., T.M., and C.S.W. Paper proofreading and approval: all authors.

DECLARATION OF INTERESTS

The authors declare no conflicts of interest.

Received: January 25, 2024
Revised: April 4, 2024
Accepted: July 16, 2024
Published: July 31, 2024

REFERENCES

1. Bhatt, S., Weiss, D.J., Cameron, E., Bisanzio, D., Mappin, B., Dalrymple, U., Battle, K., Moyes, C.L., Henry, A., Eckhoff, P.A., et al. (2015). The effect of malaria control on Plasmodium falciparum in Africa between 2000 and 2015. Nature *526*, 207–211.
2. WHO (2022). World Malaria Report 2022 (World Health Organization).
3. Antonio-Nkondjio, C., Sonhafouo-Chiana, N., Ngadjeu, C.S., Doumbe-Belisse, P., Talipouo, A., Djamouko-Djonkam, L., Kopya, E., Bamou, R., Awono-Ambene, P., and Wondji, C.S. (2017). Review of the evolution of insecticide resistance in main malaria vectors in Cameroon from 1990 to 2017. Parasites Vectors *10*, 1–14.
4. Tchouakui, M., Mugenzi, L.M.J., D Menze, B., Khaukha, J.N.T., Tchapga, W., Tchoupo, M., Wondji, M.J., and Wondji, C.S. (2021). Pyrethroid resistance aggravation in Ugandan malaria vectors is reducing bednet efficacy. Pathogens *10*, 415.
5. Mugenzi, L.M.J., Akosah-Brempong, G., Tchouakui, M., Menze, B.D., Tekoh, T.A., Tchoupo, M., Nkemngo, F.N., Wondji, M.J., Nwaefuna, E.K., Osae, M., and Wondji, C.S. (2022). Escalating pyrethroid resistance in two major malaria vectors Anopheles funestus and Anopheles gambiae (sl) in Atatam, Southern Ghana. BMC Infect. Dis. *22*, 799.
6. Hemingway, J. (2017). The way forward for vector control. Science *358*, 998–999.
7. Ranson, H., N'guessan, R., Lines, J., Moiroux, N., Nkuni, Z., and Corbel, V. (2011). Pyrethroid resistance in African anopheline mosquitoes: what are the implications for malaria control? Trends Parasitol. *27*, 91–98. https://doi.org/10.1016/j.pt.2010.08.004.
8. Riveron, J.M., Tchouakui, M., Mugenzi, L., Menze, B.D., Chiang, M.-C., and Wondji, C.S. (2018). Insecticide Resistance in Malaria Vectors: An Update at a Global Scale. In Towards Malaria Elimination - A Leap Forward, S. Manguin and V. Dev, eds. (IntechOpen).
9. Hemingway, J., Hawkes, N.J., McCarroll, L., and Ranson, H. (2004). The molecular basis of insecticide resistance in mosquitoes. Insect Biochem. Mol. Biol. *34*, 653–665.
10. Weill, M., Malcolm, C., Chandre, F., Mogensen, K., Berthomieu, A., Marquine, M., and Raymond, M. (2004). The unique mutation in ace-1 giving high insecticide resistance is easily detectable in mosquito vectors. Insect Mol. Biol. *13*, 1–7.
11. Kreppel, K.S., Viana, M., Main, B.J., Johnson, P.C.D., Govella, N.J., Lee, Y., Maliti, D., Meza, F.C., Lanzaro, G.C., and Ferguson, H.M. (2020). Emergence of behavioural avoidance strategies of malaria vectors in areas of high LLIN coverage in Tanzania. Sci. Rep. *10*, 14527.
12. Balabanidou, V., Kampouraki, A., MacLean, M., Blomquist, G.J., Tittiger, C., Juárez, M.P., Mijailovsky, S.J., Chalepakis, G., Anthousi, A., Lynd, A., and Antoine, S. (2016). Cytochrome P450 associated with insecticide resistance catalyzes cuticular hydrocarbon production in Anopheles gambiae *113*, 9268–9273.
13. Ranson, H., and Lissenden, N. (2016). Insecticide resistance in African Anopheles mosquitoes: a worsening situation that needs urgent action to maintain malaria control. Trends Parasitol. *32*, 187–196.
14. Hollingworth, R.M., and Gadelhak, G.G. (1998). Mechanisms of action and toxicity of new pesticides that disrupt oxidative phosphorylation. Rev. Toxicol. *2*, 253–266.
15. Huang, P., Yan, X., Yu, B., He, X., Lu, L., and Ren, Y. (2023). A Comprehensive Review of the Current Knowledge of Chlorfenapyr: Synthesis, Mode of Action, Resistance, and. Molecules *28*, 7673.
16. Ihara, M., Buckingham S.D., Matsuda, K. & Sattelle D.B. 24, 2925-2934 (2017).
17. Agossa, F.R., Padonou, G.G., Koukpo, C.Z., Zola-Sahossi, J., Azondekon, R., Akuoko, O.K., Ahoga, J., N'dombidje, B., Akinro, B., Fassinou, A.J.Y., and Sezonlin, M. (2018). Efficacy of a novel mode of action of an indoor residual spraying product, SumiShield® 50WG against susceptible and resistant populations of Anopheles gambiae (sl) in Benin. Parasit & Vectors *11*, 1–13.
18. Fongnikin, A., Houeto, N., Agbevo, A., Odjo, A., Syme, T., N'Guessan, R., and Ngufor, C. (2020). Efficacy of Fludora® Fusion (a mixture of deltamethrin and clothianidin) for indoor residual spraying against pyrethroid-resistant malaria vectors: laboratory and experimental hut evaluation. Parasit & Vectors *13*, 1–11.
19. Accrombessi, M., Cook, J., Ngufor, C., Sovi, A., Dangbenon, E., Yovogan, B., Akpovi, H., Hounto, A., Thickstun, C., Padonou, G.G., et al. (2021). Assessing the efficacy of two dual-active ingredients long-lasting insecticidal nets for the control of malaria transmitted by pyrethroid-resistant vectors in Benin: study protocol for a three-arm, single-blinded, parallel, cluster-randomized controlled trial. BMC Infect. Dis. *21*, 194. https://doi.org/10.1186/s12879-021-05879-1.
20. Mosha, J.F., Kulkarni, M.A., Messenger, L.A., Rowland, M., Matowo, N., Pitt, C., Lukole, E., Taljaard, M., Thickstun, C., Manjurano, A., et al. (2021). Protocol for a four parallel-arm, single-blind, cluster-randomised trial to assess the effectiveness of three types of dual active ingredient treated nets compared to pyrethroid-only long-lasting insecticidal nets to prevent malaria transmitted by pyrethroid insecticide-resistant vector mosquitoes in Tanzania. BMJ Open *11*, e046664. https://doi.org/10.1136/bmjopen-2020-046664.
21. Tungu, P.K., Michael, E., Sudi, W., Kisinza, W.W., and Rowland, M. (2021). Efficacy of interceptor® G2, a long-lasting insecticide mixture net treated with chlorfenapyr and alpha-cypermethrin against Anopheles funestus: experimental hut trials in north-eastern Tanzania. Malar. J. *20*, 180.
22. Tchouakui, M., Thiomela, R.F., Nchoutpouen, E., Menze, B.D., Ndo, C., Achu, D., Tabue, R.N., Njiokou, F., Joel, A., and Wondji, C.S. (2023). A

Chlorfenapyr-Based Net Interceptor® G2 Shows High Efficacy Against a Pyrethroid Resistant Anopheles funestus from Central Cameroon.

23. Kibondo, U.A., Odufuwa, O.G., Ngonyani, S.H., Mpelepele, A.B., Matanilla, I., Ngonyani, H., Makungwa, N.O., Mseka, A.P., Swai, K., Ntabaliba, W., and Stutz, S. (2022). Influence of testing modality on bioefficacy for the evaluation of Interceptor(®) G2 mosquito nets to combat malaria mosquitoes in Tanzania. Parasites Vectors *15*, 124. https://doi.org/10.1186/s13071-022-05207-9.

24. Tchouakui, M., Thiomela, R.F., Nchoutpouen, E., Menze, B.D., Ndo, C., Achu, D., Tabue, R.N., Njiokou, F., Joel, A., and Wondji, C.S. (2023). High efficacy of chlorfenapyr-based net Interceptor® G2 against pyrethroid-resistant malaria vectors from Cameroon. Infect. Dis. Poverty *12*, 81. https://doi.org/10.1186/s40249-023-01132-w.

25. Riveron, J. M. 7, 1819-1832 (2017).

26. Djuicy, D.D., Hearn, J., Tchouakui, M., Wondji, M.J., Irving, H., Okumu, F.O., and Wondji, C.S. (2020). CYP6P9-Driven Signatures of Selective Sweep of Metabolic Resistance to Pyrethroids in the Malaria Vector *Anopheles funestus* Reveal Contemporary Barriers to Gene Flow. Genes *11*, 1314.

27. Weedall, G.D., Mugenzi, L.M.J., Menze, B.D., Tchouakui, M., Ibrahim, S.S., Amvongo-Adjia, N., Irving, H., Wondji, M.J., Tchoupo, M., Djouaka, R., et al. (2019). A cytochrome P450 allele confers pyrethroid resistance on a major African malaria vector, reducing insecticide-treated bednet efficacy. Sci. Transl. Med. *11*, eaat7386.

28. Mugenzi, L.M.J., Menze, B.D., Tchouakui, M., Wondji, M.J., Irving, H., Tchoupo, M., Hearn, J., Weedall, G.D., Riveron, J.M., and Wondji, C.S. (2019). Cis-regulatory CYP6P9b P450 variants associated with loss of insecticide-treated bed net efficacy against Anopheles funestus. Nat. Commun. *10*, 4652.

29. Mugenzi, L.M., Menze, B.D., Tchouakui, M., Wondji, M.J., Irving, H., Tchoupo, M., Hearn, J., Weedall, G.D., Riveron, J.M., Cho-Ngwa, F., and Wondji, C.S. (2020). A 6.5 kb intergenic structural variation enhances P450-mediated resistance to pyrethroids in malaria vectors lowering bed net efficacy. Mol. Ecol. *29*, 4395–4411.

30. Yunta, C., Ooi, J.M.F., Oladepo, F., Grafanaki, S., Pergantis, S.A., Tsakireli, D., Ismail, H.M., and Paine, M.J.I. (2023). Chlorfenapyr metabolism by mosquito P450s associated with pyrethroid resistance identifies potential activation markers. Sci. Rep. *13*, 14124.

31. Menze, B.D., Tchouakui, M., Mugenzi, L.M.J., Tchapga, W., Tchoupo, M., Wondji, M.J., Chiumia, M., Mzilahowa, T., and Wondji, C.S. (2022). Marked aggravation of pyrethroid resistance in major malaria vectors in Malawi between 2014 and 2021 is partly linked with increased expression of P450 alleles. BMC Infect. Dis. *22*, 660.

32. Tchouakui, M., Assatse, T., Tazokong, H.R., Oruni, A., Menze, B.D., Nguiffo-Nguete, D., Mugenzi, L.M.J., Kayondo, J., Watsenga, F., Mzilahowa, T., et al. (2023). Detection of a reduced susceptibility to chlorfenapyr in the malaria vector Anopheles gambiae contrasts with full susceptibility in Anopheles funestus across Africa. Sci. Rep. *13*, 2363.

33. Oxborough, R.M., Seyoum, A., Yihdego, Y., Chabi, J., Wat'senga, F., Agossa, F.R., Coleman, S., Musa, S.L., Faye, O., Okia, M., et al. (2021). Determination of the discriminating concentration of chlorfenapyr (pyrrole) and Anopheles gambiae sensu lato susceptibility testing in preparation for distribution of Interceptor® G2 insecticide-treated nets. Malar. J. *20*, 1–10.

34. Hunt, D.A., and Treacy, M. (1998). Insecticides with Novel Modes of Action: Mechanisms and Application (Springer), pp. 138–151.

35. Black, B.C., Hollingworth, R.M., Ahammadsahib, K.I., Kukel, C.D., and Donovan, S. (1994). Insecticidal action and mitochondrial uncoupling activity of AC-303,630 and related halogenated pyrroles. Pestic. Biochem. Physiol. *50*, 115–128.

36. Stica, C., Jeffries, C.L., Irish, S.R., Barry, Y., Camara, D., Yansane, I., Kristan, M., Walker, T., and Messenger, L.A. (2019). Characterizing the molecular and metabolic mechanisms of insecticide resistance in Anopheles gambiae in Faranah, Guinea. Malar. J. *18*, 244.

37. Meiwald, A., Clark, E., Kristan, M., Edi, C., Claire, L., Jeffries, C.L., Pelloquin, B., Irish, S.R., Walker, T., Louisa, A., and Messenger, L.A. (2020). Reduced long-lasting insecticidal net efficacy and pyrethroid insecticide resistance are associated with over-expression of CYP6P4, CYP6P3 and CYP6Z1 in populations of Anopheles coluzzii from South-East Cote d Ivoire. Preprint at bioRxiv. https://doi.org/10.1101/2020.09.24.311639.

38. Ngufor, C., Fongnikin, A., Rowland, M., and N'Guessan, R. (2017). Indoor residual spraying with a mixture of clothianidin (a neonicotinoid insecticide) and deltamethrin provides improved control and long residual activity against pyrethroid resistant Anopheles gambiae sl in Southern Benin. PLoS One *12*, e0189575.

39. Romero, A., and Anderson, T.D. (2016). High levels of resistance in the common bed bug, Cimex lectularius (Hemiptera: Cimicidae), to neonicotinoid insecticides. J. Med. Entomol. *53*, 727–731.

40. Mazíková, V., Sroková, I., and Ebringerová, A. (2009). Solvent-free synthesis and properties of carboxymethyl starch fatty acid ester derivatives. Chem. Pap. *63*, 71–76.

41. Lees, R., Praulins, G., Davies, R., Brown, F., Parsons, G., White, A., Ranson, H., Small, G., and Malone, D. (2019). A testing cascade to identify repurposed insecticides for next-generation vector control tools: screening a panel of chemistries with novel modes of action against a malaria vector. Gates Open Res. *3*, 1464.

42. Tchouakui, M., Assatse, T., Mugenzi, L.M.J., Menze, B.D., Nguiffo-Nguete, D., Tchapga, W., Kayondo, J., Watsenga, F., Manzambi, E.Z., Osae, M., and Wondji, C.S. (2022). Comparative study of the effect of solvents on the efficacy of neonicotinoid insecticides against malaria vector populations across Africa. Infect. Dis. Poverty *11*, 35.

43. WHO (2022). Manual for Monitoring Insecticide Resistance in Mosquito Vectors and Selecting Appropriate Interventions (World Health Organization).

44. N'Guessan, R., Odjo, A., Ngufor, C., Malone, D., and Rowland, M. (2016). A Chlorfenapyr Mixture Net Interceptor(R) G2 Shows High Efficacy and Wash Durability against Resistant Mosquitoes in West Africa. PLoS One *11*, e0165925. https://doi.org/10.1371/journal.pone.0165925.

45. Ngufor, C., Fagbohoun, J., Critchley, J., N'Guessan, R., Todjinou, D., Malone, D., Akogbeto, M., and Rowland, M. (2017). Which intervention is better for malaria vector control: insecticide mixture long-lasting insecticidal nets or standard pyrethroid nets combined with indoor residual spraying? Malar. J. *16*, 340. https://doi.org/10.1186/s12936-017-1987-5.

46. Tungu, P.K., Michael, E., Sudi, W., Kisinza, W.W., and Rowland, M. (2021). Efficacy of interceptor(R) G2, a long-lasting insecticide mixture net treated with chlorfenapyr and alpha-cypermethrin against Anopheles funestus: experimental hut trials in north-eastern Tanzania. Malar. J. *20*, 180. https://doi.org/10.1186/s12936-021-03716-z.

47. Mugenzi, L.M.J., A Tekoh, T., S Ibrahim, S., Muhammad, A., Kouamo, M., Wondji, M.J., Irving, H., Hearn, J., and Wondji, C.S. (2023). The duplicated P450s CYP6P9a/b drive carbamates and pyrethroids cross-resistance in the major African malaria vector Anopheles funestus. PLoS Genet. *19*, e1010678.

48. Sheppard, C.D., and Joyce, J.A. (1998). Increased susceptibility of pyrethroid-resistant horn flies (Diptera: Muscidae) to chlorfenapyr. J. Econ. Entomol. *91*, 398–400.

49. Pimprale, S.S., Besco, C.L., Bryson, P.K., and Brown, T.M. (1997). Increased susceptibility of pyrethroid-resistant tobacco budworm (Lepidoptera: Noctuidae) to chlorfenapyr. J. Econ. Entomol. *90*, 49–54.

50. Hearn, J., Djoko Tagne, C.S., Ibrahim, S.S., Tene-Fossog, B., Mugenzi, L.M.J., Irving, H., Riveron, J.M., Weedall, G.D., and Wondji, C.S. (2022). Multi-omics analysis identifies a CYP9K1 haplotype conferring pyrethroid resistance in the malaria vector Anopheles funestus in East Africa. Mol. Ecol. *31*, 3642–3657.

51. Hunt, R.H., Brooke, B.D., Pillay, C., Koekemoer, L.L., and Coetzee, M. (2005). Laboratory selection for and characteristics of pyrethroid resistance in the malaria vector Anopheles funestus. Med. Vet. Entomol. *19*, 271–275.

CellPress
OPEN ACCESS

52. Morgan, J.C., Irving, H., Okedi, L.M., Steven, A., and Wondji, C.S. (2010). Pyrethroid resistance in an Anopheles funestus population from Uganda. PLoS One *5*, e11872.

53. Tchouakui, M., Assatse, T., Mugenzi, L.M., Menze, B.D., Nguiffo-Nguete, D., Tchapga, W., Kayondo, J., Watsenga, F., Manzambi, E.Z., Osae, M., and Wondji, C.S. (2022). Comparative study of the effect of solvents on the efficacy of neonicotinoid insecticides against malaria vector populations across Africa. Infectious Diseases of Poverty *11*, 23–31.

54. Assatse, T., Tchouakui, M., Mugenzi, L., Menze, B., Nguiffo-Nguete, D., Tchapga, W., Kekeunou, S., and Wondji, C.S. (2023). *Anopheles funestus* Populations across Africa Are Broadly Susceptible to Neonicotinoids but with Signals of Possible Cross-Resistance from the GSTe2 Gene. Trav. Med. Infect. Dis. *8*, 244.

55. WHO (2016). Test Procedures for Insecticide Resistance Monitoring in Malaria Vector Mosquitoes (World Health Organization).

56. WHO (2013). Guidelines for Laboratory and Field Testing of Long-Lasting Insecticidal Nets (WHO Library Cataloguing-in-Publication Data).

57. Menze, B.D., Kouamo, M.F., Wondji, M.J., Tchapga, W., Tchoupo, M., Kusimo, M.O., Mouhamadou, C.S., Riveron, J.M., and Wondji, C.S. (2020). An Experimental Hut Evaluation of PBO-Based and Pyrethroid-Only Nets against the Malaria Vector *Anopheles funestus* Reveals a Loss of Bed Nets Efficacy Associated with *GSTe2* Metabolic Resistance. Genes *11*, 143.

58. Riveron, J.M. (2013). USA *110*, 252–257.

59. Blandin, S., Moita, L.F., Köcher, T., Wilm, M., Kafatos, F.C., and Levashina, E.A. (2002). Reverse genetics in the mosquito Anopheles gambiae: targeted disruption of the Defensin gene. EMBO Rep. *3*, 852–856.

60. Kouamo, M.F.M., Ibrahim, S.S., Hearn, J., Riveron, J.M., Kusimo, M., Tchouakui, M., Ebai, T., Tchapga, W., Wondji, M.J., Irving, H., et al. (2021). Genome-Wide Transcriptional Analysis and Functional Validation Linked a Cluster of Epsilon Glutathione S-Transferases with Insecticide Resistance in the Major Malaria Vector *Anopheles funestus* across Africa. Genes *12*, 561.

61. Pritchard, M.P., Ossetian, R., Li, D.N., Henderson, C.J., Burchell, B., Wolf, C.R., and Friedberg, T. (1997). A General Strategy for the Expression of Recombinant Human Cytochrome P450s inEscherichia coliUsing Bacterial Signal Peptides: Expression of CYP3A4. CYP2A6, and CYP2E1 *345*, 342–354.

62. Ibrahim, S.S., Riveron, J.M., Bibby, J., Irving, H., Yunta, C., Paine, M.J.I., and Wondji, C.S. (2015). Allelic Variation of Cytochrome P450s Drives Resistance to Bednet Insecticides in a Major Malaria Vector. PLoS Genet. *11*, e1005618.

63. Badolo, A., Guelbéogo, W.M., Tiono, A.B., Traoré, A., Sagnon, N., and Sirima, S.B. (2014). Laboratory evaluation of Fendona 6SC® treated bednets and Interceptor® long-lasting nets against Anopheles gambiae sl in Burkina Faso. Parasitol. Res. *113*, 1069–1075.

64. Chouaibou, M., Simard, F., Chandre, F., Etang, J., Darriet, F., and Hougard, J.M. (2006). Efficacy of bifenthrin-impregnated bednets against Anopheles funestus and pyrethroid-resistant Anopheles gambiae in North Cameroon. Malar. J. *5*, 77. https://doi.org/10.1186/1475-2875-5-77.

65. Menze, B.D., Kouamo, M.F., Wondji, M.J., Tchapga, W., Tchoupo, M., Kusimo, M.O., Mouhamadou, C.S., Riveron, J.M., and Wondji, C.S. (2020). An Experimental Hut Evaluation of PBO-Based and Pyrethroid-Only Nets against the Malaria Vector Anopheles funestus Reveals a Loss of Bed Nets Efficacy Associated with GSTe2 Metabolic Resistance. Genes *11*, 143.

66. Lowry, R. (2004). VassarStats : Website for Statistical Computation (Vassar College).

STAR★METHODS

KEY RESOURCES TABLE

REAGENT or RESOURCE	SOURCE	IDENTIFIER
Bacterial and virus strains		
E. coli JM109 competent cells	Promega, United Kingdom	Cat# P9751
Biological samples		
Mosquitoes RNA and cDNA	Our lab	NA
Mosquitoes DNA	Our lab	NA
Chemicals, peptides, and recombinant proteins		
DH5α competent cells	Thermo Scientific (MA, USA)	Cat#18265017
pACYC-184	ATCC (USA)	Cat# 37033
Clothianidin	Sigma Aldrich, (Hilden, Germany)	Cat# 33589
Chlorfenapyr	Sigma Aldrich (Hilden, Germany)	Cat# 37913
Potassium Phosphate monobasic	Sigma Aldrich (Hilden, Germany)	Cat# 7778-77-0
Potassium Phosphate dibasic	Sigma Aldrich (Hilden, Germany)	Cat# 7758-11-4
Leupeptin hemisulfate	Thermo Scieintfic (MA, USA)	Cat# J61188.MC
Glucose-6-phosphate dehydrogenase	Sigma Aldrich (Hilden, Germany)	Cat# G7877
5-aminolevulinic acid hydrochloride (ALA)	Sigma Aldrich (Hilden, Germenay)	Cat# A3785
Cytochrome P450 Reductase	Our lab expression	NA
Cytochrome P450	Our lab expression	NA
Cytochrome *b*5	Our lab Expression	NA
Isopropyl B-D-1-thiogalactopyranoside (IPTG)	Sigma Aldrich (Hilden, Germany)	Cat# I6758
Aprotinin from bovine lung	Sigma Aldrich	Cat# 9087-70-1
Acetonitrile HPLC grade	Agilent Technologies	EC# 200-835-2
Water HPLC grade	Agilent Technologies	EC# 5191-5120
EDTA	Promega, United Kingdom	Cat#V4231
D-glucose 6 phosphate sodium salt	Sigma Aldrich (Hilden, Germany)	Cat#54010-71-8
Critical commercial assays		
PicoPure RNA isolation kit	Arcturus	KIT0204
Super-Script III for CDNA synthesis	Invitrogen	18080051
QIAquick® Gel Extraction Kit	QIAGEN	28704
QIAprep® Spin Miniprep Kit	QIAGEN	27104
MEGAscript™ RNAi-Kit	ThermoFisher	AM1626
Deposited data		
Raw and analyzed data	This paper	NA
Experimental models: Organisms/strains		
FANG strain	CRID insectary	N.A
FUMOZ strain	CRID insectary	N.A
Anopheles funestus from Malawi	CRID insectary	N.A
Oligonucleotides		
Forward Primer for genotyping of *CYP6Pa* marker	TCCCGAAATACAGCCTTTCAG	N.A
Reverse Primer for genotyping of *CYP6Pa* marker	ATTGGTGCCATCGCTAGAAG	
Forward Primer for genotyping of *CYP6Pb* marker	CCCCCACAGGTGGTAACTATCTGAA	
Reverse Primer for genotyping of *CYP6Pb* marker	TTATCCGTAACTCAATAGCGATG	

(*Continued on next page*)

CellPress
OPEN ACCESS

Continued

REAGENT or RESOURCE	SOURCE	IDENTIFIER
Restriction enzyme for *CYP6Pa:* Taq I (cut site, 5′-TCGA-3′)	New England Biolabs	Cat# R0149S
Restriction enzyme for *CYP6Pb:* NmuCl (Tsp45I) (cut site 5′-GTSAC-3′)	New England Biolabs	Cat# R0583S
Software and algorithms		
Prism Graphpad 8.0	N/A	NA

RESOURCE AVAILABILITY

Lead contact

Further information and requests for resources and reagents should be directed to and will be fulfilled by the lead contact, Dr. Magellan Tchouakui, magellan.tchouakui@crid-cam.net.

Materials availability

This study did not generate new or unique reagents.

Data and code availability

- All the relevant datasets supporting the conclusions of this article are included within the article.
- This paper does not report original code
- Any additional information required to reanalyze the data reported in this work paper is available from the lead contact upon request.

EXPERIMENTAL MODEL AND STUDY PARTICIPANT DETAILS

Trials design

The huts were built following the prototype recommended by WHO for the West African region as previously described. The hut was constructed on a concrete base of cement surrounded by a drain channel to trap aunt. The walls were made from concrete bricks and plastered inside and outside with a plaster made from a mixture of cement and sand, the roof was made from corrugated iron and the ceiling was made from plywood. In the context of this trial since we released mosquitoes in the huts all the windows opening were closed to avoid mosquitoes escaping.

Anopheles funestus strain used

Both field and laboratory samples of An. funestus were used in this study. Field mosquitoes were collected in Southern Africa [(Malawi (MWI), Chikwawa (16°1′ S, 34°47′ E) in 2021[31] and reared at CRID insectary, in Cameroon. The laboratory strain was the FANG/FUMOZ (FG/FZ) hybrid colony resulting from the crossing between FANG, a completely insecticide-susceptible colony originating from Angola, and the FUMOZ, a pyrethroid-resistant colony from Southern Africa Mozambique.[51] The crossing between FANG and FUMOZ created a heterogeneous population segregating the genotypes of duplicated CYP6P9a_R, and CYP6P9b_R variants which are closed to fixation in Chikwawa, completely fixed in FUMOZ, but absent in FANG.[27,28] Blood-fed and gravid female mosquitoes were collected indoor in houses in Chikwawa using electric aspirators. Fully gravid females were introduced into 1.5mL Eppendorf tubes to lay eggs using the forced-egg laying protocol.[52]

Ethical approval

The national and regional (Center region) ethics committee for health research of CaMEROon approved the protocol of the study (ID: 2021/07/1372/CE/CNERSH/SP and CE No: 0803/CRERSHC/2021). Written, informed and signed consent was obtained from sleepers before starting the trials. All methods were performed per the relevant guidelines and regulations.

METHOD DETAILS

Assessing the impact of *CYP6P9a_R/-b_R* on the efficacy of chlorfenapyr and clothianidin using CDC bottle assay

The F_1 adult female mosquitoes from field and F_3/F_4 generation of the hybrid FG/FZ were used to assess clothianidin and CFP susceptibility patterns in CDC bottle assays using established protocols[32,53,54] after establishing their resistance profiles to the previously WHO-recommended insecticides in WHO-tube tests.[55] Approximately 24h after coating bottles with insecticide, 25 females (2–5 d old) were exposed to 150 μg/ml clothianidin (using acetone alone as solvent) or 4 μg/ml (using acetone + MERO (81% rapeseed oil methyl ester; manufactured by Bayer CropScience) as solvent) for 1h, and the knocked-down were recorded at the end of the

60min (Kd-60), and mortality at 24h or 7d, respectively acetone + MERO as solvent and for acetone only as solvent. For CFP, we first established the LC_{50} (with 10, 20, 30, 50, and 100 μg/mL CFP), using the hybrid strain, FG/FZ, and the LT_{50} (using 100 μg/mL CFP with varying exposure time, from 10 to 60min). Experiments were repeated with LC_{50}/LT_{50} until enough alive and dead mosquitoes were obtained and genotyped[27,28] for *CYP6P9a_R/-b_R* markers, allowing us to establish their impact on the ability of mosquitoes to survive CFP and CLTD exposures.

Assessing the impact of *CYP6P9a_R/-b_R* on the efficacy of chlorfenapyr-based nets using tunnel tests

Comparative tunnel assays were performed using: i. Control net (bed nets without insecticide); ii. Interceptor net (200 mg/m^2 Alpha-cypermethrin); iii. Interceptor G2 (100 mg/m^2 Alpha-cypermethrin +200 mg/m^2 chlorfenapyr); and iv. In-house, custom made impregnated chlorfenapyr-only net (100 mg/m^2 and 200 mg/m^2 chlorfenapyr) to establish the impact of CYP6P9a/-b on the efficacy of CFP-based nets. On average 100 mosquitoes (sugar-starved for 1h), aged 5–8 days were released in the long section of the glass tunnel at 06:00 p.m. with a guinea pig bait positioned on the other side of the net so that mosquitoes must pass through the holed net to access the bait. The following morning, between 06:00 and 09:00a.m., mosquitoes were removed (separately from each section of the tunnel) using a mouth aspirator, counted, and scored as alive or dead, blood-fed or unfed, after which they were held for 72h to evaluate the final mortality. The main outcome measures were 12 h mortality, 72h post-exposure mortality, and blood-feeding inhibition.[56] The impact of CYP6P9a/-b on the efficacy of the nets was evaluated by comparing the genotype and allele frequency for these markers between dead and alive, as well as blood-fed vs. unfed.

Assessing the impact of *CYP6P9a_R/-b_R* on the efficacy of CFP-based nets using experimental hut trials

The experimental hut trial (EHTs) was performed in Elende (3°41′57.27″N, 11°33′28.46’’E), a rural village in central CaMEROon, close to Yaoundé where we recently built 12 experimental West Africa type huts (42) made of concrete bricks to confirm the impact of the duplicated genes on the efficacy of various nets. The study was carried out with the FG/FZ hybrid strain against the following net treatments: i. Control; ii. Interceptor; iii. Interceptor G2; and iv. In-house, custom made impregnated chlorfenapyr-only net (100 mg/m^2 chlorfenapyr), to better capture the effect of resistance markers on the CFP net without pyrethroid).

The F_3 generation of the hybrid strain was released in the huts with each treatment for one week as previously described[27,28,57]. Mosquitoes were released in the hut in the evening and collected early in the morning using glass tubes from the room (the floor, walls, and roof of the hut), inside the bed net, and from the exit traps in the veranda. Each compartment of the hut had its bag to avoid mixing samples. Surviving mosquitoes were provided with sugar solution and held for 72 h in paper cups after which delayed mortality was assessed. Samples were recorded in observation sheets as dead/blood-fed, alive/blood-fed, dead/unfed, and alive/unfed. The effect of each treatment was expressed relative to the control (untreated net) by assessing induced exophily (the proportion of mosquitoes that exited early through the exit traps because of the treatment); the mortality rate, an indicator of the potential killing effect of the insecticide-treated bed nets; and the rate of blood feeding, an indicator of insecticide resistance and personal protection. To establish the impact of the *CYP6P9a*/b-mediated metabolic resistance to pyrethroids on the effectiveness of various nets, the PCR-RFLP diagnostic assay[27,28] was used to genotype a subset of each treatment including the dead, alive, blood-fed, and unfed mosquitoes. Odds Ratio and Fisher’s exact test were used to assess the impact of CYP6P9a_R and CYP6P9b_R on the ability of mosquitoes to survive and blood feed after exposure to insecticide-treated bed nets.

Assessing the impact of *CYP6P9a_R/-b_R* on the efficacy of clothianidin-based IRS using experimental hut trials

To establish the impact of *CYP6P9a/-b* on the efficacy of CLTD-based IRS, hut trials was also conducted. The F_3 hybrid strain of FG/FZ crosses were released in 4 experimental huts with the following insecticide treatments: i. Unsprayed hut (control); ii. Deltamethrin sprayed at 25 mg/m^2; clothianidin sprayed at 200 mg/m^2; and Fludora Fusion sprayed at 25 mg/m^2 of deltamethrin +200 mg/m^2 of clothianidin. Mosquitoes were released in the treated huts in the evening, and they were collected early in the morning using glass tubes, from the rooms, and from the exit traps on the veranda. Surviving mosquitoes were provided with sugar solution and held for 72 h in paper cups after which delayed mortality was assessed. Samples were recorded in observation sheets as alive/dead and blood-fed/unfed and the CYP6P9a_R and CYP6P9b_R markers were genotyped on the respective phenotype to establish their impact on the efficacy of these CLTD-based IRS.

Assessing the impact of CYP6P9a/-b on efficacy of chlorfenapyr and clothianidin using *in vivo* transgenic expression in with *Drosophila melanogaster*

To further assess the role of CYP6P9a/-b on the efficacy of CFP/CLTD against *An. funestus*, transgenic D. melanogaster expressing CYP6P9a or CYP6P9b were used to screen for resistance phenotype, to validate if over-expression of each of these genes alone can confer resistance to these insecticides, as previously done for permethrin and deltamethrin.[58] Two transgenic lines, UAS-*CYP6P9a* and UAS-*CYP6P9b* were generated, with injection, balancing and crosses conducted as was done in our previous studies. Ubiquitous expression of each transgene in adult F_1 progeny (experimental group) was obtained after crossing virgin females from the driver strain Act5C-GAL4 ["y [1] w [*]; P(Act5C-GAL4-w) E1/CyO","1; 2"] (Bloomington Stock Center, IN, USA) with UAS-CYP6P9a/b males. Similarly, adult F_1 control progeny (control group) with the same genetic background as the experimental group but without CYP6P9a or -b insert was obtained by crossing virgin females from the driver strain Act5C-GAL4 and UAS recipient line males (which do

not carry the pUASattB-*CYP6P9a* or -b insertion). These different lines were comparatively exposed to chlorfenapyr (10 μg/ml) and clothianidin (50 μg/ml) and the mortality was monitored up to 24 h.

Assessing the impact of CY6P9a/-b on efficacy of chlorfenapyr and clothianidin using RNA-interference

To further validate the influence of *CYP6P9a/b* clothianidin and chlorfenapyr resistance, RNAi knockdown approach was also exploited. Gene-specific primers for double-stranded RNA (dsRNA) synthesis were designed with BLOCK-iT RNAi Designer (Thermo Fisher Scientific, UK). The dsRNA was synthesised using an *in vitro* Transcription T7 MEGAscript RNAi-Kit (for siRNA Synthesis) (Thermo Fisher Scientific, UK) following the manufacturer's instructions. The dsRNA of the green fluorescent protein gene was also synthesised and utilised as a negative control.[59,60] NanoDrop 2000 spectrophotometer was used to measure the concentration and the purity of dsRNA samples, and RNA quality was established by 2% agarose gel electrophoresis, and dsRNA samples was stored at − 20°C until use. A total of 3 μg dsRNA (0.69nL) was injected into 2–5 days old female mosquitoes using a Nanoject II micro-injector (Drummond, Burton, OH, USA). The injected mosquitoes were used for susceptibility testing 4 days post-injection of ds-RNA or dsGFP, using bottles treated with either 10μg chlorfenapyr or 150μg clothianidin (acetone only as solvent) following the protocol described above.[43] Following exposure, they were transferred to paper cups and supplied with sugar then mortality was recorded up to 7 days post-exposure. Each RNAi treatment was replicated four times and each replicate comprised 20–25 mosquitoes.

Investigating chlorfenapyr and clothianidin metabolising activity of *CYP6P9a* using *in vitro* protein expression and metabolism assays

Expression plasmid was created by fusing the *CYP6P9a* cDNA NH_2-terminus in frame with the P450 initiation codon to a fragment from a bacterial ompA+2 leader sequence with its downstream ala-pro linker, following established protocols.[61,62] This P450 construct was cloned into the expression vector pCW-ori+ and co-expressed as membrane proteins in *E. coli JM109*, together with *An. funestus* cytochrome P450 reductase (*AfCPR*). Strategy for cloning of *AfCPR* into the expression pACYC-184 followed a modified protocol of Pritchard and colleagues[61] with details to be provided in a separate publication (in preparation). Metabolism assays were conducted with clothianidin, and chlorfenapyr following protocols described previously.[62] The assay was carried out in 0.1 M potassium phosphate buffer (KPi, pH 7.4) and NADPH regeneration buffer, which were added to the bottom of chilled 1.5 mL tubes. Membranes expressing the recombinant CYP6P9a and AfCPR, were added to the side of the tube, to which *An. gambiae* cytochrome b_5 was also reconstituted in a ratio 1:4. These were pre-incubated for 5 min at 30°C, with shaking at 1,200 rpm, before adding 20 μM of clothianidin or chlorfenapyr, with continuous shaking at 1,200 rpm and 30°C for 1.5 h. Reactions were quenched with 0.1 mL ice-cold acetonitrile and incubated for 5 min at 1200 rpm. Tubes were centrifuged at 16,000 rpm and 4°C for 15 min, and 150 μL of supernatant transferred into HPLC vials. For clothianidin,100 μL samples were injected into isocratic mobile phase (15:85% acetonitrile to water containing 0.1% phosphoric acid), with column temperature set to 40°C, detection wavelength set to 254 nm, and a flow rate set to 1 mL/min. Similar approach was used for chlorfenapyr, but with mobile phase comprised of 80:20% acetonitrile to water, column temperature set at 35°C, and detection wavelength set to 210 nm. Peaks were separated with a 250 mm C18 column (Acclaim 120, Dionex) on an Agilent 1260 Infinity HPLC machine (Agilent, city, country). All reactions were carried out in triplicate with experimental samples (+NADPH containing the NADPH regeneration buffer) and negative controls (-NADPH not containing NADP+ in the regeneration buffer). Enzyme activity was calculated as percentage depletion (difference in the amount of clothianidin and chlorfenapyr remaining in the +NADPH tubes compared with the –NADPH), and a student's t-test was used to estimate significance.

QUANTIFICATION AND STATISTICAL ANALYSIS

Experimental hut trial

To calculate the proportion of each entomological outcomes and the level of significance between the treatments and between the control for each entomological outcomes, the XLSTAT software was used as done previously.[27,63–65]

Test of association between the P450 genes (*CYP6P9a/-b*) and the entomological outcomes

To investigate the association between the P450 alleles and mosquito's ability to survive, blood feed or exit the room with bed nets, VassarStats[66] was used to estimate the odds ratio based on a fisher exact probability test with a 2x2 contingency table as previously described.[27,65] All the proportions were compared using Chi-square test.

Cell Reports, Volume *43*

Supplemental information

Substrate promiscuity of key resistance P450s confers clothianidin resistance while increasing chlorfenapyr potency in malaria vectors

Magellan Tchouakui, Sulaiman S. Ibrahim, Mersimine K. Mangoua, Riccado F. Thiomela, Tatiane Assatse, Sonia L. Ngongang-Yipmo, Abdullahi Muhammad, Leon J.M. Mugenzi, Benjamin D. Menze, Themba Mzilahowa, and Charles S. Wondji

Supplemental files

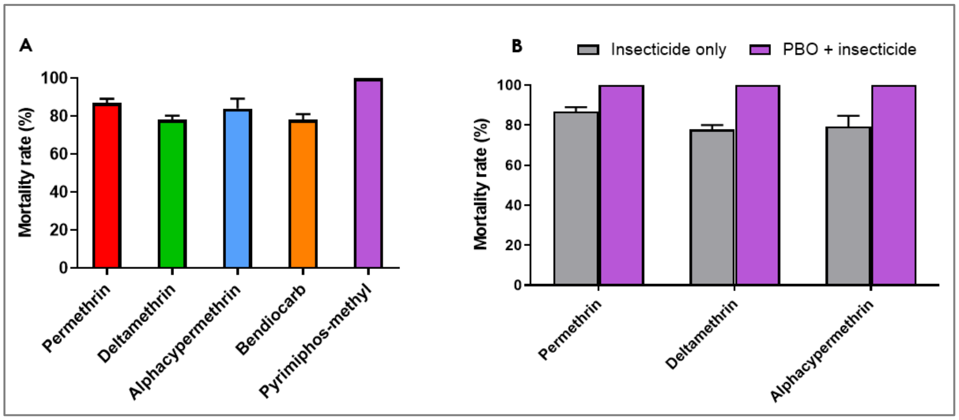

Figure S1: Susceptibility profile of the hybrid strain FG/FZ F_3 to the four main classes of insecticides recommended by WHO: A) Mortality rate after exposure to pyrethroids (permethrin, deltamethrin and alpha-cypermethrin), carbamate (bendiocarb) and organophosphate (pirimiphos-methyl) following 60-min exposure; B) PBO synergist assays with pyrethroids; Data are shown as mean ± SEM.

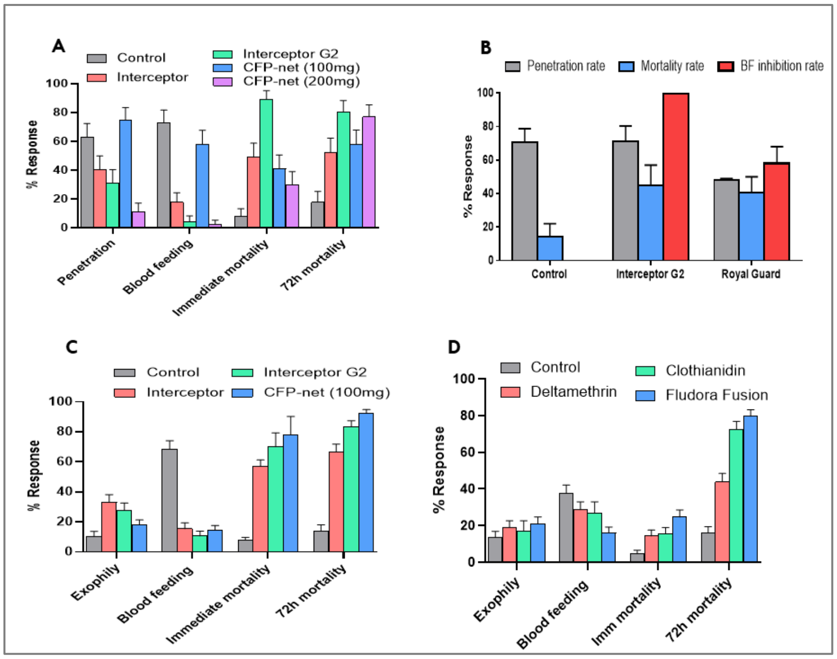

Figure S2: Efficacy of chlorfenapyr and clothianidin-based control tools against *An. funestus* in tunnel assays and experimental hut trials: A) Efficacy of CFP-based nets compared to pyrethroid-only net on the hybrid strain FG/FZ in tunnel tests; B) Efficacy of interceptor G2 and Royal Guard nets on *An. funestus* from Malawi in tunnel tests; C) Efficacy of CFP-based nets compared to pyrethroid-only net on the hybrid strain FG/FZ in EHT; D) Efficacy of the CLTD-based IRS formulations compared to pyrethroid-only IRS product in EHT. Data are shown as mean ± 95% CI.

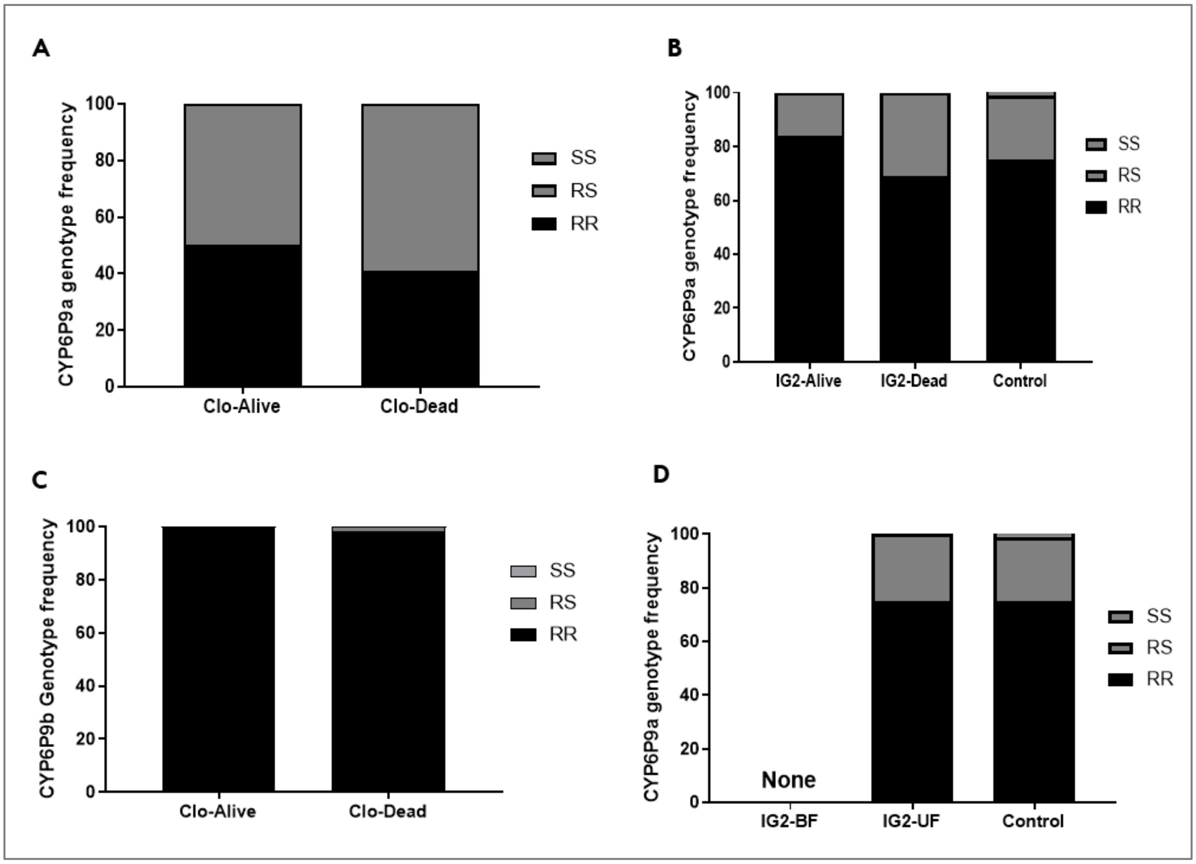

Figure S3: Impact of *CYP6P9a and CYP6P9b* on the ability of field *An. funestus* from Malawi to survive clothianidin and chlorfenapyr exposure. Genotype distribution of CYP6P9a (A) and CYP6P9b (C) between alive and dead after exposure to clothianidin in CDC bottle assays. Genotype distribution of CYP6P9a between dead and alive (B), and blood-fed/unfed (B) after exposure to the CFP-based net IG2 in tunnel test.

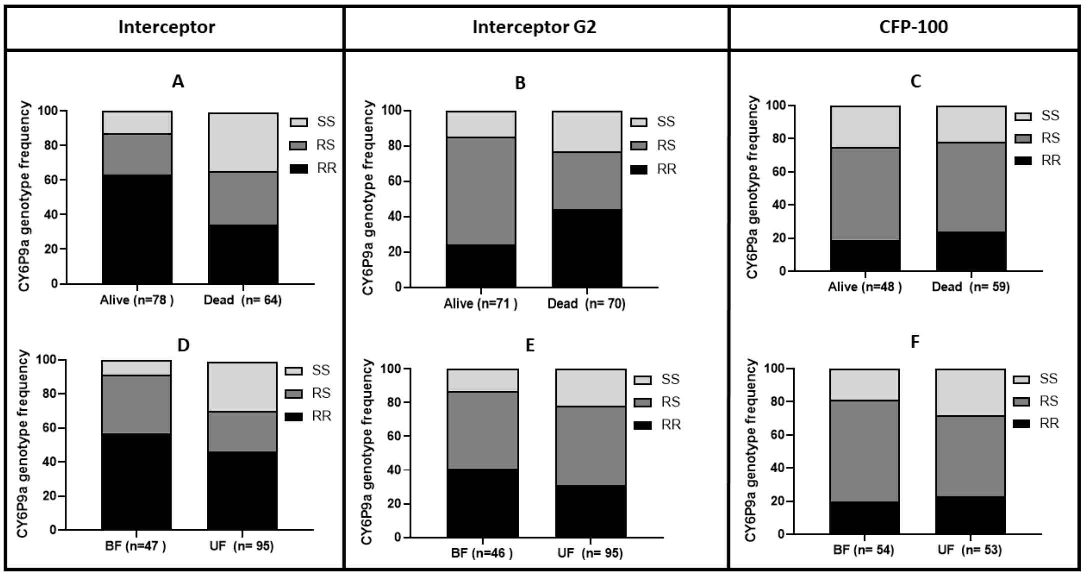

Figure S4: Impact of the *CYP6P9a* on the efficacy of CFP-based nets on the hybrid strain FG/FZ after tunnel tests . Genotype distribution between alive and dead after exposure to Interceptor (A), Interceptor G2 (B) and CFP-100 (C); Genotype distribution between blood fed and unfed after exposure to Interceptor (D), Interceptor G2 (E) and CFP-100 (F);

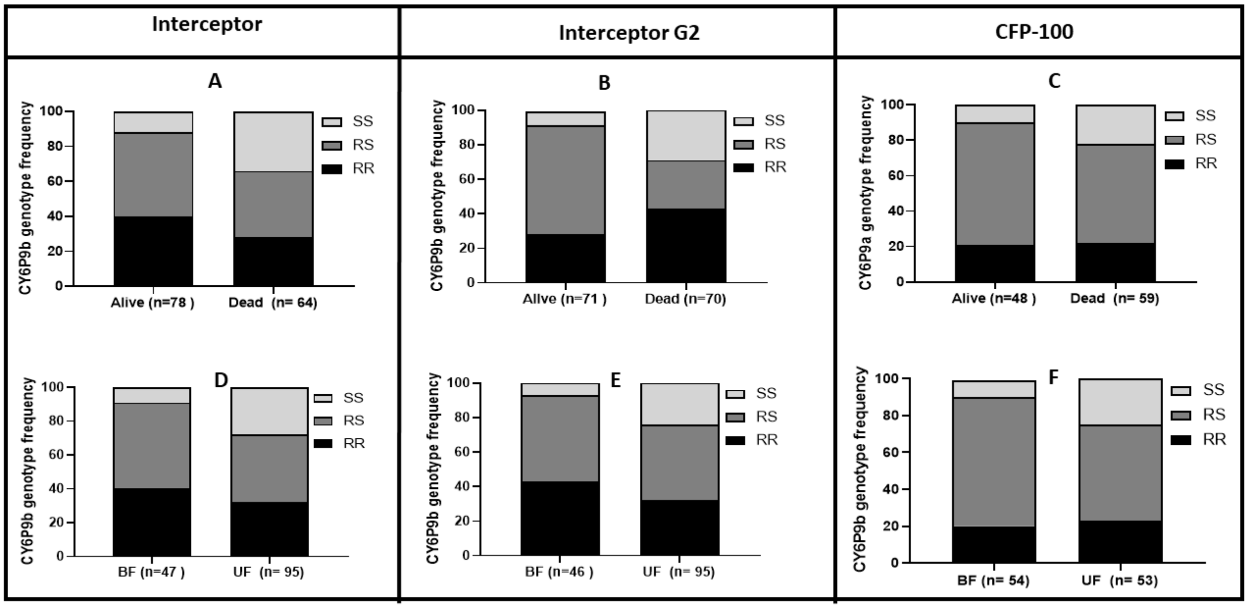

Figure S5: Impact of the C*YP6P9b* on the efficacy of CFP-based nets on the hybrid strain FG/FZ after tunnel tests. Genotype distribution between alive and dead after exposure to Interceptor (A), Interceptor G2 (B) and CFP-100 (C); Genotype distribution between blood fed and unfed after exposure to Interceptor (D), Interceptor G2 (E) and CFP-100 (F);

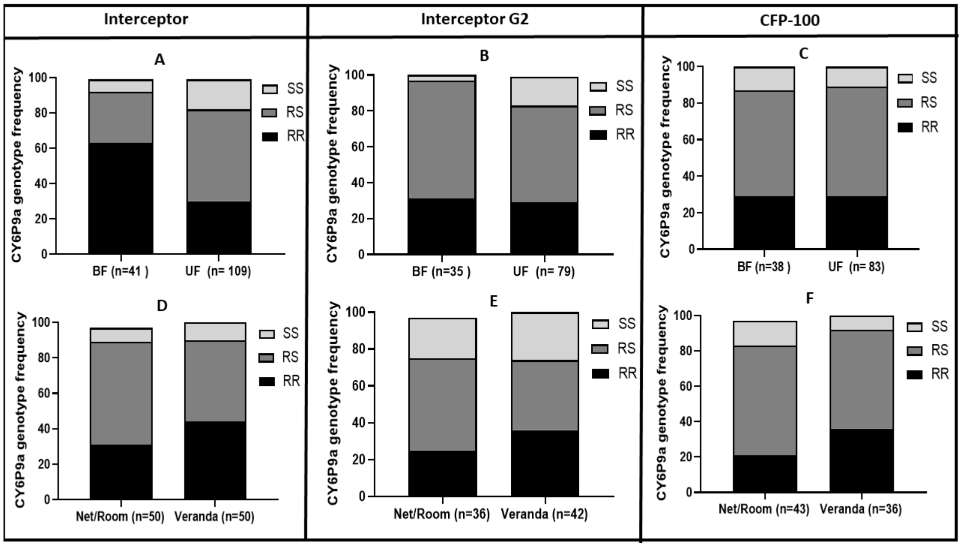

Figure S6: Impact of the C*YP6P9a* on the efficacy of CFP-based nets on the hybrid strain FG/FZ in EHT. Genotype distribution between blood-fed and unfed after exposure to Interceptor (A), Interceptor G2 (B) and CFP-100 (C); Genotype distribution between indoor (Net/Room) and outdoor (veranda) after exposure to Interceptor (D), Interceptor G2 (E) and CFP-100 (F).

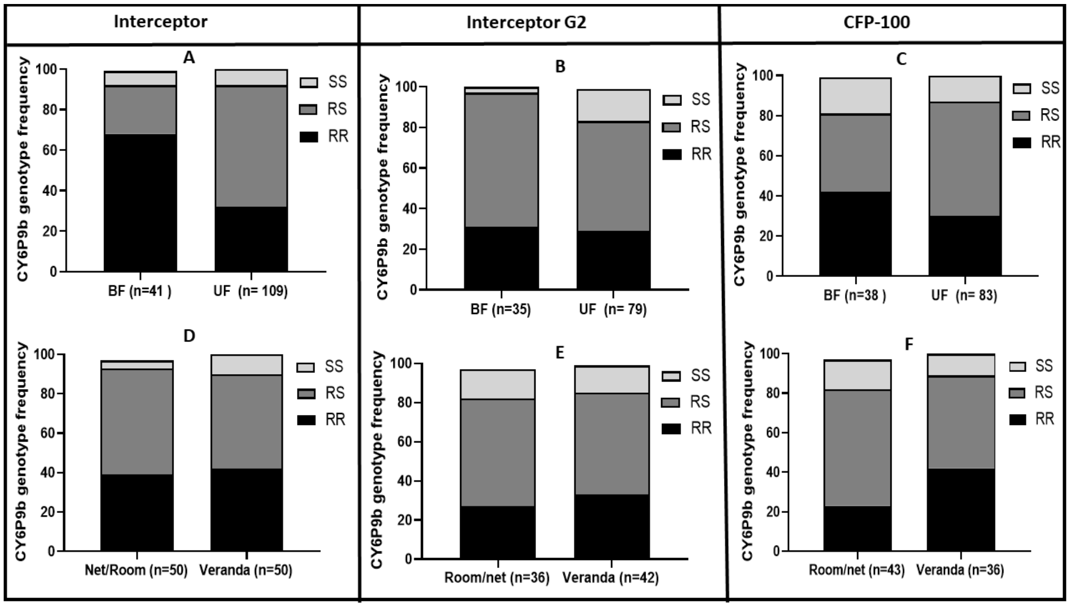

Figure S7: Impact of the *CYP6P9b* on the efficacy of CFP-based nets on the hybrid strain FG/FZ in EHT. Genotype distribution between blood-fed and unfed after exposure to Interceptor (A), Interceptor G2 (B) and CFP-100 (C); Genotype distribution between indoor (Net/Room) and outdoor (veranda) after exposure to Interceptor (D), Interceptor G2 (E) and CFP-100 (F).

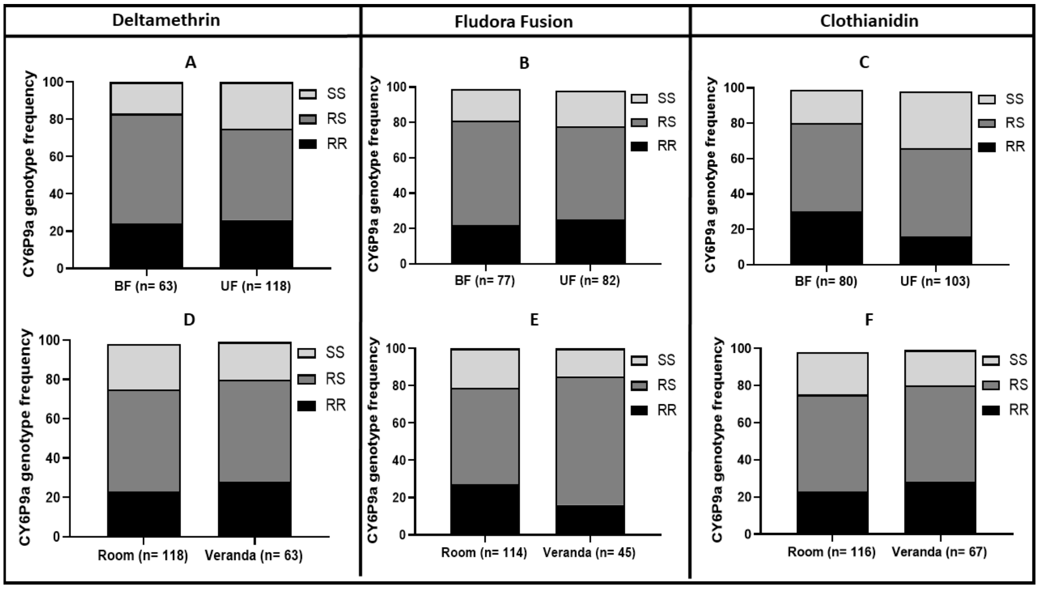

Figure S8: Impact of the *CYP6P9a* on the efficacy of clothianidin-based IRS formulation on the hybrid strain FG/FZ in EHT. Genotype distribution between blood-fed and unfed after exposure to deltamethrin (A), Fludora Fusion (B) and clothianidin (C); Genotype distribution between indoor (Room) and outdoor (veranda) after exposure to deltamethrin (D), Fludora Fusion (E) and clothianidin (F).

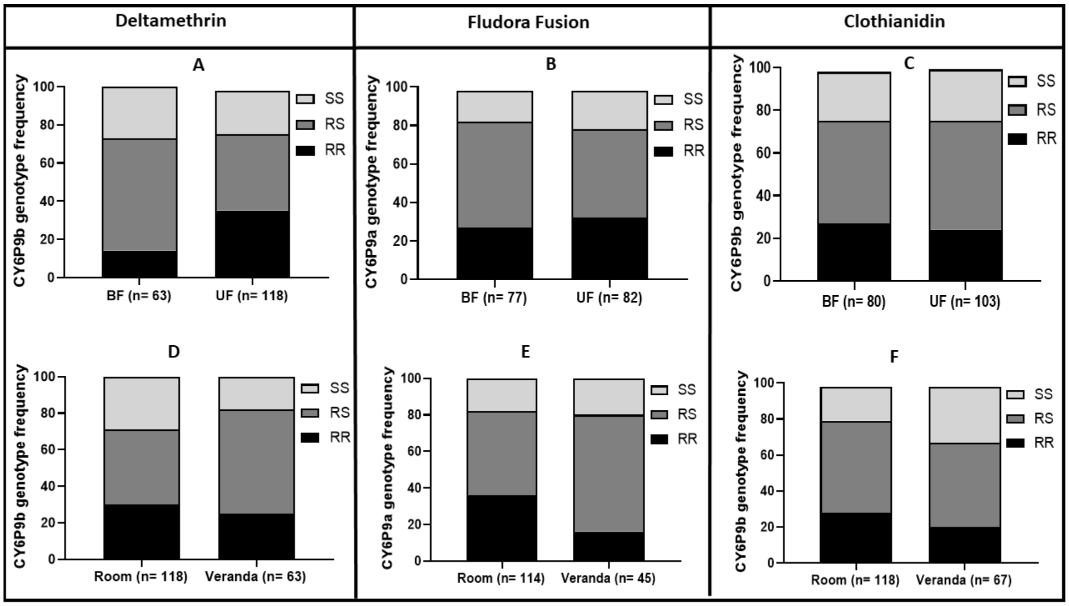

Figure S9: Impact of the *CYP6P9b* on the efficacy of clothianidin-based IRS formulation on the hybrid strain FG/FZ in EHT. Genotype distribution between blood-fed and unfed after exposure to deltamethrin (A), Fludora Fusion (B) and clothianidin (C); Genotype distribution between indoor (Room) and outdoor (veranda) after exposure to deltamethrin (D), Fludora Fusion (E) and clothianidin (F).

Table S1: Correlation between genotypes of the CYP6P9a/b and ability of the hybrid strain FG/FZ to survive CFP exposure in CDC bottle assays

	Odd-Ratio	Confidence interval	*P*-value
	**Genotypes at *CYP6P9a* locus**		
**RR vs SS**	0.1	0.06 - 0.3	<0.0001
**RS vs SS**	0.7	0.3 - 1.7	0.7
**RR vs RS**	0.1	0.09 - 0.3	<0.0001
**R vs S**	0.7	0.4 - 1.3	0.4
	**Genotypes at *CYP6P9b* locus**		
**RR vs SS**	0.2	0.1 - 0.5	0.0003
**RS vs SS**	0.7	0.3 -1.5	0.09
**RR vs RS**	0.3	0.2 - 0.7	0.002
**R vs S**	0.6	0.9 - 2.7	0.1

OR: odd-ratio; CI: confidence interval; RR: homozygote resistant; RS: heterozygote; SS: homozygote susceptible.

Table S2: Impact of CYP6P9a and b on the ability of the hybrid strain FG/FZ to survive clothianidin exposure in CDC bottle assay

	Odd-Ratio	Confidence interval	*P*-value
	**Genotypes at *CYP6P9a* locus**		
**RR vs SS**	7.5	2.1-27.5	0.001
**RS vs SS**	3.5	1.1-11.5	0.02
**RR vs RS**	2.1	0.9-5.1	0.06
**R vs S**	2.2	1.2-3.8	0.005
	**Genotypes at *CYP6P9b* locus**		
**RR vs SS**	7.1	1.9-26.1	0.002
**RS vs SS**	3	0.9-9.9	0.05
**RR vs RS**	2.4	0.9-5.6	0.04
**R vs S**	2.2	1.2-3.8	0.005

OR: odd-ratio; CI: confidence interval; RR: homozygote resistant; RS: heterozygote; SS: homozygote susceptible.

Table S3: Performance of clothianidin-based IRS against F_3 hybrid FG/FZ *An. funestus* mosquitoes

Outcomes	Control	Deltamethrin (25 mg/m2)	Clothianidin (200 mg/m2)	Fludora® Fusion (225 mg/m2)
females caught	339	447	408	471
%Exophily (95% CI)	13.67 (10.45-16.88) [a]	19.02 (15.38-22.65) [b]	17.16 (13.50-20.82) [b]	21.02 (17.34-24.70) [b]
%Blood fed (95% CI)	37.59 (33.05-42.12) [c]	28.64 (24.44-32.83) [b]	26.72 (22.42-31.01) [b]	15.92 (12.62-19.23) [a]
% Immediate mortality (95% CI)	4.56 (2.61-6.51) [a]	14.32 (11.07-17.56) [b]	15.44 (11.93-18.95) [b]	24.63 (20.74-28.52) [c]
%Mortality (72 h) (95% CI)	15.95 (12.52-19.37) a	43.85 (39.25-48.45) [b]	72.55 (68.22-76.88) [c]	79.62 (75.98-83.26) [c]

Table S4: Correlation between genotypes at the *CYP6P9a/b* locus and the ability to survive/blood feed in the presence of various nets in tunnel tests

Genotype combination	Interceptor			Interceptor G2			CFP-100		
	OR	CI	*P*-value	OR	CI	*P*-value	OR	CI	*P*-value
	**Impact of *CYP6P9a* mortality**								
**RR vs SS**	123	18-1273	<0.0001	1.7	0.7-4.6	0.3	0.8	0.3-1.9	0.8
**RS vs SS**	66	10-684	<0.0001	1.1	0.5-2.3	0.8	2.2	1.2-4.5	0.01
**RR vs RS**	1.8	0.9-3.8	0.1	1.5	0.7-3.3	0.2	0.3	0.1-0.8	0.02
**R vs S**	4.3	2.3-7.6	<0.0001	1.2	0.7-2.2	0.4	1.1	0.6-1.8	0.8
	**Impact of *CYP6P9b* mortality**								
**RR vs SS**	4.04	1.8-8.7	0.0006	2.3	0.9-5.7	0.08	2.1	0.8-5.1	0.1
**RR vs RS**	1.1	0.5-2.1	0.7	0.2	0.1-0.5	0.0002	0.7	0.3-1.5	0.4
**RS vs SS**	3.5	1.5-8.04	0.001	8.1	3.2-19.4	<0.0001	2.7	1.1-6.1	0.01
**R vs S**	2.0	1.1-3.4	0.02	1.1	0.6-1.9	0.7	1.2	0.6-2.1	0.5
	**Impact of *CYP6P9a* on blood feeding success**								
**RR vs SS**	0.8	0.4-1.6	0.7	1.3	0.7- 2.4	0.3	0.6	0.3-1.4	0.3
**RS vs SS**	3.9	1.7-9.7	0.001	2.2	0.9-5.3	0.06	1.2	0.5- 2.9	0.6
**RR vs RS**	4.5	1.8-10.6	0.0008	1.6	0.7-3.7	0.2	1.8	0.9-3.6	0.1
**R vs S**	2.06	1.1-3.6	0.02	1.5	0.8-2.6	0.1	1.1	0.6-2.06	0.6
	**Impact of *CYP6P9b* on blood feeding success**								
**RR vs SS**	3.8	1.5-8.9	0.002	4.6	1.7-11.4	0.0013	2.4	0.9-6.1	0.09
**RR vs RS**	0.9	0.5-1.8	>0.9	1.1	0.6- 2.1	0.6	0.6	0.3-1.2	0.2
**RS vs SS**	3.9	1.6-9.2	0.001	3.8	1.5-9.7	0.0035	3.7	1.6-9.1	0.002
**R vs S**	1.7	1-3.1	0.06	1.8	1-3.2	0.05	1.3	0.7-2.3	0.3

OR: odd-ratio; CI: confidence interval; CFP: chlorfenapyr; RR: homozygote resistant; RS: heterozygote; SS: homozygote susceptible.

Table S5: Correlation between genotypes at the CYP6P9a/b locus and the ability to survive exposure/blood feed in the presence of various nets in experimental huts

Genotype combination	Interceptor OR	Interceptor CI	Interceptor P-value	Interceptor G2 OR	Interceptor G2 CI	Interceptor G2 P-value	CFP-100 OR	CFP-100 CI	CFP-100 P-value
	**Impact of *CYP6P9a* mortality**								
**RR vs SS**	47,5	7,6-497,8	<0,0001	13,2	2,2-44,3	0,003	0,3	0,1-1,3	0,1
**RS vs SS**	27,1	4,5-285,4	<0,0001	25,00	3,9-265,6	<0,0001	3,5	1,3-9,7	0,01
**RR vs RS**	1,7	0,9-3,1	0,09	0,5	0,2-0,9	0,05	0,1	0,04-0,2	<0,0001
**R vs S**	3,0	1,6-5,7	0,0004	1,7	1,-3,06	0,06	0,6	0,3-1,1	0,2
	**Impact of *CYP6P9b* mortality**								
**RR vs SS**	25,2	4,2-272,4	<0,0001	13,2	2,2-144,3	0,003	2,2	0,7-6,4	0,2
**RR vs RS**	2,2	1,2-4,04	0,008	0,5	0,2-0,9	0,05	0,6	0,3-1,1	0,1
**RS vs SS**	11,2	1,6-121,3	0,004	25	3,9-265,6	<0,0001	3,5	1,3-9,5	0,01
**R vs S**	2,4	1,3-4,6	0,005	1,2	0,7-2,2	0,4	1,1	0,6-1,9	0,7
	**Impact of *CYP6P9a* on blood feeding success**								
**RR vs SS**	5,1	1,8-12,7	0,0009	5,7	1,6-19,6	0,007	0,8	0,3-2,1	0,8
**RS vs SS**	1,3	0,5-3,4	0,6	6,5	1,8- 21,7	0,002	0,8	0,3-1,9	0,8
**RR vs RS**	3,7	1,9-7,1	<0,0001	0,8	0,4-1,6	0,7	1,03	0,5-1,9	>0,9
**R vs S**	2,7	1,5-5,2	0,001	1,3	0,7-2,4	0,3	0,9	0,5-1,712	>0,9
	**Impact of *CYP6P9b* on blood feeding success**								
**RR vs SS**	2,4	0,76,6	0,1	5,7	1,6-19,60	0,007	1,01	0,4-2,3	>0,9
**RR vs RS**	5,3	2,7-9,7	<0,0001	0,8	0,4-1,6	0,7	2,04	1,07-3,7	0,02
**RS vs SS**	0,4	0,1-1,3	0,2	6,5	1,8-21,7	0,002	0,4	0,2-1,09	0,1
**R vs S**	2,4	1,2-4,5	0,007	1,3	0,7-2,4	0,3	1,1	0,6-2,04	0,6

OR: odd-ratio; CI: confidence interval; CFP: chlorfenapyr; RR: homozygote resistant; RS: heterozygote; SS: homozygote susceptible.

Table S6: Correlation between genotypes at the CYP6P9a/b locus and the ability to survive exposure/blood feed in the presence of various IRS treatments in experimental huts

Genotype combination	Deltamethrin			Clothianidin			Fludora		
	OR	CI	*P*-value	OR	CI	P-value	OR	CI	*P*-value
	**Impact of *CYP6P9a* mortality**								
**RR vs SS**	2.5	1.3-4.7	0.001	0.7	0.4-1.5	0.2	1.2	0.6-2.3	0.3
**RS vs SS**	11.4	3.2-41.3	<.0001	1.1	0.5-2.4	0.5	2	0.8-5.2	0.1
**RR vs RS**	4.4	1.2-16.4	0.01	1.4	0.7-3.0	0.1	1.6	0.7-3.9	0.2
**R vs S**	3.4	1.7-6.5	0.0001	1	0.6-1.8	0.5	1.3	0.7-2.3	0.2
	**Impact of *CYP6P9b* on mortality**								
**RR vs SS**	11.4	3.2-41.3	<.0001	1.5	0.5-2.4	0.3	1.1	0.8-5.2	0.1
**RR vs RS**	2.5	1.3-4.7	0.001	0.7	0.4-1.5	0.6	1.2	0.6-2.3	0.3
**RS vs SS**	4.4	1.2-16.4	0.01	2.05	0.7-3.0	0.05	1.6	0.7-3.9	0.2
**R vs S**	3.4	1.7-6.5	0.0001	1	0.6-1.8	0.5	1.3	0.7-2.3	0.2
	**Impact of *CYP6P9a* on blood-feeding success**								
**RR vs SS**	0.2	0.1-0.4	<.0001	1.2	0.6-2.3	0.4	0.4	0.2-0.7	**0.004**
**RS vs SS**	0.3	0.1-0.8	0.02	1.6	0.7-3.6	0.1	0.7	0.3-1.8	0.3
**RR vs RS**	1.5	0.5-4.15	0.6	1.4	0.7-2.8	0.7	2	0.8-4.6	0.7
**R vs S**	0.3	0.2-0.6	0.0007	0.7	0.4-1.4	0.2	0.7	0.4-1.4	0.2
	**Impact of *CYP6P9b* on blood-feeding success**								
**RR vs SS**	0.3	0.1-0.8	0.01	1.6	0.7-3.6	0.1	1.4	0.3-1.8	0.3
**RR vs RS**	0.27	0.1-0.6	<.0001	1.2	0.6-2.3	0.4	0.7	0.2-0.7	**0.004**
**RS vs SS**	1.2	0.6-2.4	0.6	0.98	0.7-2.8	0.9	2	0.8-4.6	0.7
**R vs S**	0.3	0.2-0.6	0.0007	0.7	0.4-1.4	0.2	0.7	0.4-1.4	0.2

OR: odds ratio; CI: confidence interval; RR: homozygote resistant; RS: heterozygote; SS: homozygote susceptible.

***Table S7:** Combined impact of CYP6P9a and CYP6P9b on the mortality and blood feeding of* the hybrid strain FG/FZ *after exposure to various nets in tunnel assays*

Interceptor						
	Mortality			Blood feeding		
	OR	CI	P-value	OR	CI	P-value
**RR/RR vs RS/RS**	1.7	0.8-3.7	0.1	0.9	0.4-2	>0.9
**RR/RR vs SS/SS**	8.06	2.7-20.7	<0.0001	3.2	1.2-7.6	0.01
**RR/RR vs SS/RR**	0.6	0.1-2.8	0.6	5.3	0.7-66.7	0.1
**RR/RR vs SS/RS**	0.3	0.02-2.08	0.3	4	0.5-53.1	0.3
**RS/RS vs SS/SS**	4.6	1.6-12.1	0.004	3.3	1.2-8.2	0.01
**RS/RS vs RS/SS**	1.4	0.15-21.1	>0.9	2.7	0.29-40.6	0.5
**RS/RS vs RR/SS**	7.1	0.8-86.4	0.08	0.1	0.01-1.2	0.1
**Interceptor G2**						
	OR	CI	P-value	OR	CI	P-value
**RR/RR vs RS/RS**	0.1	0.06-0.3	<0.0001	1.6	0.7-3.3	0.2
**RR/RR vs SS/SS**	0.8	0.32.4	>0.9	5.9	1.6-17.4	0.002
**RR/RR vs SS/RR**	0.2	0.01-5.6	0.4	1.1	0.05-23.2	>0.9
**RR/RR vs SS/RS**	0.03	0.003-0.2	0.0006	0.1	0.01-1.002	0.07
**RS/RS vs SS/SS**	5.5	2.07-13.7	0.0004	3.6	1.1-10.7	0.02
**RS/RS vs RS/SS**	1.6	0.08-32.7	>0.9	0.7	0.03-14.4	>0.9
**RS/RS vs RR/SS**	0.1	0.01-1	0.1	0.9	0.1-4.6	>0.9
**CFP-100**						
	OR	CI	P-value	OR	CI	P-value
**RR/RR vs RS/RS**	0.9	0.4-1.9	0.8	0.6	0.3-1.3	0.3
**RR/RR vs SS/SS**	1.7	0.6-4.7	0.3	2.5	0.8-7.01	0.1
**RR/RR vs SS/RR**	1.7	0.1-27.2	>0.9	1.5	0.16-23.03	>0.9
**RR/RR vs SS/RS**	0.05	0.005-0.4	0.001	0.1	0.02-0.6	0.02
**RS/RS vs SS/SS**	1.9	0.8-4.5	0.1	4	1.6-10.02	0.002
**RS/RS vs RR/SS**	/	/	/	1.7	0.19-25.3	>0.9

Table S8: *Combined impact of CYP6P9a and CYP6P9b on the mortality and blood feeding of* the hybrid strain FG/FZ *after exposure to various nets in EHT*

**Interceptor**						
	**Mortality**			**Blood feeding**		
	**OR**	**CI**	**P-value**	**OR**	**CI**	**P-value**
**RR/RR vs RS/RS**	1.8	1.01-3.5	0.05	4.8	2.4-9.6	<0.0001
**RR/RR vs SS/SS**	25.04	4.06-271.2	<0.0001	2.1	0.7-6.3	0.2
**RR/RR vs SS/RS**	25.04	4-271.2	<0.0001	21.7	3.2-240	0.0002
**RS/RS vs SS/SS**	13.3	2.2-145	0.002	0.4	0.1-1.3	0.2
**RS/RS vs RS/SS**	0.9	0.05-18.9	>0.9	/	/	/
**RS/RS vs RR/SS**	/	/	/	2.2	0.1-43.05	0.5
**Interceptor G2**						
	**OR**	**CI**	**P-value**	**OR**	**CI**	**P-value**
**RR/RR vs RS/RS**	0.50	0.2-0.97	0.05	2.5	1.2-5.03	0.01
**RR/RR vs SS/SS**	13.8	2.2-151.2	0.003	1.1	0.4-3.2	0.8
**RR/RR vs SS/RR**	1.6	0.1-24.5	>0.9	4.9	0.6-65.8	0.2
**RR/RR vs SS/RS**	2.4	0.6-9.07	0.3	0.5	0.1-1.9	0.3
**RS/RS vs SS/SS**	27.2	4.2-290	<0.0001	0.4	0.1-1.1	0.1
**CFP-100**						
	**OR**	**CI**	**P-value**	**OR**	**CI**	**P-value**
**RR/RR vs RS/RS**	0.1	0.05-0.3	<0.0001	1.3	0.6-2.7	0.4
**RR/RR vs SS/SS**	0.4	0.1-1.5	0.3	0.8	0.3-2.1	0.7
**RR/RR vs SS/RS**	0.2	0.01-4.8	0.3	0.2	0.02-2.04	0.3
**RS/RS vs SS/SS**	3.02	1.03-8.7	0.04	0.6	0.2-1.5	0.3
**RS/RS vs RS/SS**	6.04	0.9-74.9	0.1	0.2	0.04-1.2	0.1
**RS/RS vs RR/SS**	0.6	0.03-12.8	>0.9	4.9	0.7-65.5	0.2
